# Impact of sex and socioeconomic status on the likelihood of surgery, hospitalization, and use of medications in inflammatory bowel disease: a systematic review and meta-analysis

**DOI:** 10.1186/s13643-024-02584-3

**Published:** 2024-06-24

**Authors:** Nathalie Fogh Rasmussen, Caroline Moos, Laura Helene Keiding Gregersen, Zainab Hikmat, Vibeke Andersen, Anders Green, Tine Jess, Gunvor Iben Madsen, Andreas Kristian Pedersen, Sofie Ronja Petersen, Lene Juel Kjeldsen

**Affiliations:** 1 Hospital Pharmacy Research Unit, Department of Regional Health Research–IRS, Hospital Sønderjylland, University Hospital of Southern Denmark, Aabenraa, Denmark; 2 National Center of Excellence for Molecular Prediction of Inflammatory Bowel Disease- PREDICT, Department of Clinical Medicine, Faculty of Medicine, Aalborg University, Copenhagen, Denmark; 3 Department of Clinical Research, Hospital Sønderjylland, University Hospital of Southern Denmark, Aabenraa, Denmark; 4 Molecular Diagnostics and Clinical Research Unit, Department of Regional Health Research–IRS, Hospital Sønderjylland, University Hospital of Southern Denmark, Aabenraa, Denmark; 5 Clincial Genome Center, Department of Clinical Research, University of Southern Denmark, Odense, Denmark; 6 Steno Diabetes Center Odense, Odense University Hospital, Odense, Denmark; 7 Department of Gastroenterology and Hepatology, Aalborg University Hospital, Aalborg, Denmark; 8 Department of Clinical Pathology, Odense University Hospital, Odense, Denmark

**Keywords:** Inflammatory bowel disease, Sex differences, Socioeconomic differences, Surgery, Hospitalization

## Abstract

**Background:**

Inflammatory bowel diseases (IBDs) are associated with high healthcare utilization. This systematic review aimed to summarize what is known about the impact of sex, income, and education on the likelihood of bowel surgery, hospitalization, and use of corticosteroids and biologics among patients with IBD.

**Methods:**

We used EMBASE, MEDLINE, CINAHL, and Web of Science to perform a systematic literature search. Pooled hazard ratios (HRs) and odds ratios (ORs) with 95% confidence intervals (CIs) were calculated using random effects meta-analysis for the impact of sex on the likelihood of surgery and hospitalization. In addition, we performed subgroup analyses of the effect of IBD type (Crohn’s disease or ulcerative colitis) and age. Finally, meta-regression was undertaken for the year of publication.

**Results:**

In total, 67 studies were included, of which 23 studies were eligible for meta-analysis. In the main meta-analysis, male sex was associated with an increased likelihood of bowel surgery (HR 1.42 (95% CI 1.13;1.78), which was consistent with the subgroup analysis for UC only (HR 1.78, 95% CI 1.16; 2.72). Sex did not impact the likelihood of hospitalization (OR 1.05 (95% CI 0.86;1.30), although the subgroup analysis revealed an increased likelihood of hospitalization in CD patients (OR 1.42, 95% CI 1.28;1.58). In 9 of 10 studies, no significant sex-based differences in the use of biologics were reported, although in 6 of 6 studies, female patients had lower adherence to biologics. In 11 of 13 studies, no significant sex-based difference in the use of corticosteroids was reported. The evidence of the impact of income and education on healthcare utilization was sparse and pointed in different directions. The substantial heterogeneity between studies was explained, in part, by differences in IBD type and age.

**Conclusions:**

The results of this systematic review indicate that male patients with IBD are significantly more likely to have surgery than female patients with IBD but are not, overall, more likely to be hospitalized, whereas female patients appear to have statistically significantly lower adherence to biologics compared to male patients. Thus, clinicians should not underestimate the impact of sex on healthcare utilization. Evidence for income- and education-based differences remains sparse.

**Systematic review registration:**

PROSPERO CRD42022315788.

**Supplementary Information:**

The online version contains supplementary material available at 10.1186/s13643-024-02584-3.

## Introduction

Inflammatory bowel diseases (IBDs), with their two main subtypes, Crohn’s disease (CD) and ulcerative colitis (UC), are chronic inflammatory diseases of the gastrointestinal tract, associated with increased health resource utilization, ongoing monitoring, long-term medication, and occasionally the need for hospitalization and surgery [1, 2].

A step-up strategy is usually applied to treat mild-to-moderate IBD, starting with 5-aminosalicylic acid (5-ASA) and escalating to glucocorticoids and/or immunosuppressants, as necessary [3, 4]. Treatment with biologics is a well-known final treatment step with approximately 50% of patients starting this treatment within 2 years of diagnosis [5]. Finally, IBD patients with severe complications may undergo surgery and/or need to be hospitalized. Ten years after diagnosis 30–70% of patients with CD and 10–30% of patients with UC will undergo intestinal surgery [6–8]. Approximately 20% and 40% of patients with UC and CD are hospitalized within 5 years of diagnosis, respectively [9].

Individual treatment is highly dependent on disease phenotype, complications, severity, and activity [1, 2]. However, socio-demographic characteristics, such as education, income level, and sex, may also affect disease outcomes, impacting healthcare utilization, access, or need, as highlighted in previous literature reviews, although with contradictive conclusions [10–16]. For example, low-income and economic deprivation have been associated with higher rates of hospitalization for patients with an IBD diagnosis [10, 11, 17, 18], but lower rates of surgery [10, 18], whereas the impact on surgery remains unclear in other studies [11]. Moreover, associations between low socioeconomic status and lower adherence to medical therapy for IBD have been found in some studies [19–21] but not in others [18, 22]. In addition, some research has reported the female sex as an independent predictor for non-adherence to biological treatment [13, 15], while others did not report any difference between male and female patients [16, 23]. The evidence that sex impacts healthcare utilization for IBD patients is likewise contradictory. In one large prospective study, sex was not identified as a predictor for surgery and cumulative medication use among IBD patients [23]. In contrast, other studies report male sex as an independent predictor for surgery [12, 24, 25], yet other studies demonstrated higher surgery rates in female patients [26, 27].

Sociodemographic differences may have consequences for the likelihood of complications and severe disease, adding to the burden of social inequality. Although previous reviews addressing this topic are available, they are either out of date or have discordant or limited results, and the impact of sex and socioeconomic status on healthcare access and utilization for IBD patients thus, remains uncertain. Defining the sociodemographic determinants affecting healthcare utilization has important clinical and societal implications, such as improving our understanding of which factors to consider in both future studies and clinical practice.

We aimed to perform a systematic review and meta-analysis of the impact of socio-demographic characteristics, including income, education, and sex, on the likelihood of bowel surgery, hospitalization, and use of corticosteroids and biologics in observational studies of patients with IBD.

## Methods

### Eligibility criteria

Eligible studies for this systematic literature review included patients (children and adults) diagnosed with IBD. Studies of socioeconomic status were only included if they measured income or educational level. Accepted study designs were peer-reviewed observational studies, including cohort, case–control, cross-sectional studies, and uncontrolled trials. Published abstracts, letters to the editor, editorials, and theses were excluded.

### Information sources and search strategy

In April 2022, Embase, MEDLINE, CINAHL, and Web of Science databases were searched systematically for observational studies examining associations between the sociodemographic factors of sex, income, and educational status and four outcomes: likelihood of bowel surgery, hospitalization, use of corticosteroids and treatment with biologics. Three search blocks were used: one for IBD combined with “and” with a block for sex and a block for income and education, respectively. In addition, a “year” filter [2012–2021] was applied to all four databases. The search was updated in February 2024 with publication dates 01.01.2022–29.02.2024.

The complete search strategy is presented in Additional file 1: Table A1. The review was reported in accordance with the Preferred Reporting Items for Systematic reviews and Meta-Analyses (PRISMA) statement [28] and registered in the International prospective register of systematic reviews (PROSPERO) [Registration ID: CRD42022315788] shortly after the searches were initiated and before the analyses were conducted.

There were minor variations in the methods between the planned protocol (as registered in PROSPERO) and the study’s methodology, i.e., the end date of searches was in February 2024 rather than January 2022. Moreover, grey literature was not searched as planned, and snowballing via reference lists of included and excluded studies was not undertaken due to the comprehensive number of studies achieved from the database searches. Hospitalization was defined as “yes” or “no” to any hospitalization regardless of the frequency of reported hospitalizations.

### Selection process and data items

Screening and data extraction were performed independently by the first author Nathalie Fogh Rasmussen (NFR) and one of three other authors: LHG, CM, and ZH, using the systematic review screening tool, Covidence [29], and articles were assigned at random between LHG, CM, and ZH. Any discrepancies were resolved by NFR and one of the three other reviewers, and a third reviewer (LHG, CM, or ZH) was involved as required.

Data were extracted from all selected studies, including author, publication year, study period, data source, study design, index event, patient age (children/adult), IBD type (CD or UC), number of patients with IBD and subgroups, independent variable (sex, income or education), primary outcomes of interest, covariates, effect estimates, and main findings.

Outcomes were included if presented as frequency and percentage (*N* (%)), relative risk (RR), odds ratio (OR), or hazard ratio (HR). Surgery was defined as any first-occurring bowel surgery. Hospitalization was defined as a hospital stay/inpatient/readmission visit for any reason but excluded outpatient/ambulatory or emergency room visits. The use of corticosteroids and biological treatment was defined as any dose or duration of the medication. Studies of adherence or discontinuation of corticosteroids and biological treatment were also included.

### Study risk of bias assessment

Quality assessment of included studies was conducted using the Newcastle–Ottawa Scale (NOS) for cohort studies [30]. Although not indicated in the PROSPERO record, GRADE (Grading of Recommendations, Assessment, Development, and Evaluations) [31] was applied to studies included in the meta-analysis to assess the certainty of the body of evidence. The certainty of the evidence was classified as high, moderate, low, or very low. Two independent investigators conducted this process (CM and NFR), and disagreements were solved by discussion.

### Effect measures and synthesis methods

Analyses were performed using R version 4.2.2 + (R markdown using metaphor and E-value package). Studies eligible for meta-analysis included studies of sex if they were similar according to the type of outcome measurement, type of effect size (HR), and study design. If the number of studies calculating HRs was < 4 for a given outcome, studies calculating OR were used instead (if the number of these studies was > / = 4). Some effect estimates were converted to ensure consistency in using the female sex as the reference level.

A random-effects (RE) model using the Hartung-Knapp-Sidik-Jonkman method [32] was chosen to calculate the average distribution of associations. Where high between-study consistency was present, a fixed effect model was applied in the subgroup analyses. Where available, adjusted effect estimates presented for each study were used. Studies with more than one outcome measure (e.g., two types of surgery) were duplicated in the meta-analysis.

Between-study heterogeneity was assessed by inspecting the forest plots and by calculating the tau-squared ${({\varvec{\tau}}}^{2})$ and the *I*-squared statistics ${(I}^{2})$ with corresponding 95% confidence intervals (CIs).

Subgroup analyses and meta-regression were undertaken to explore possible causes of heterogeneity among study results. Subgroup analysis was undertaken for the categorical variables IBD type (CD/UC), age (children/adults), and country. Meta-regression was undertaken for the continuous variable “year of publication”. Furthermore, in a sensitivity analysis, the heterogeneity between univariate studies was compared to the heterogeneity between multivariate studies to examine the causes of heterogeneity. Publication bias was evaluated using a visual inspection of funnel plots’ symmetry.

## Results

### Summary of studies

**Fig. 1:**
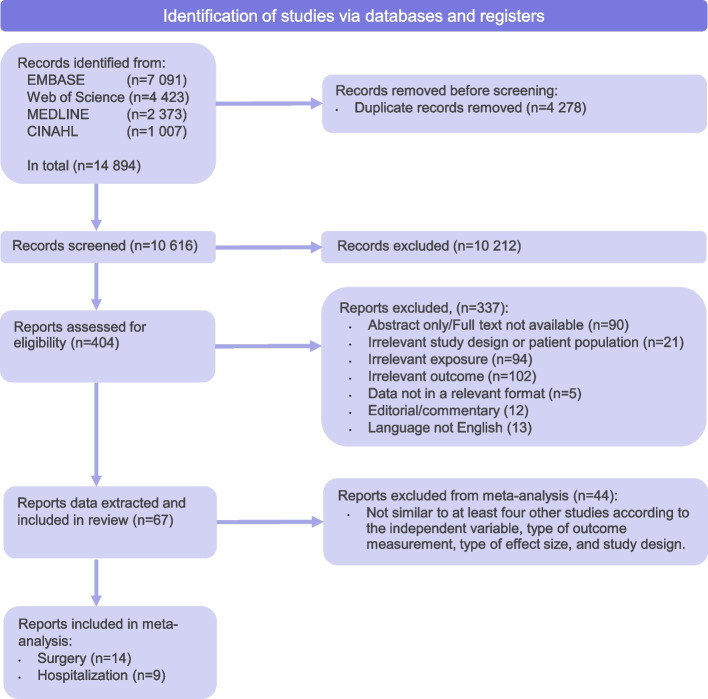
PRISMA 2020 flow diagram. *From:* Page M J, McKenzie J E, Bossuyt P M, Boutron I, Hoffmann T C, Mulrow C D et al. The PRISMA 2020 statement: an updated guideline for reporting systematic reviews *BMJ* 2021; 372:n71 doi:10.1136/bmj.n71

In total, 14,894 records were identified in the search. After removing duplicates, 10,616 records were screened, from which 404 full-texts were reviewed, and 67 studies were included in the review (Fig. 1).

Most studies were retrospective in design (73.5%) (Additional file 2: Table A2). Patients with CD were included in 77.6% of studies, and patients with UC were included in 71.6% of studies. In total, 65 (97.0%) studies reported sex, 10 (14.9%) income, and 6 (9%) education as independent variables.

In total, 23 studies were eligible for meta-analysis as they were similar according to the definition of the independent variable (only sex was comparable across studies), type of outcome measurement, type of effect size, and study design.

### Bowel surgery

A total of 41 studies (61.2%) compared the rates or frequency of bowel surgery between males and females [25, 26, 33–71]. Of these, 22 (53.7%) reported statistically significant sex-based differences in the likelihood of bowel surgery [25, 26, 33, 35, 39, 40, 44, 47, 50–53, 55, 56, 58, 62–64, 66, 67, 69, 70] (Table 1). Of these 22 studies, 19 reported that males had a higher likelihood of bowel surgery than females.

**Table 1:** Reported relative risk estimates and key findings in studies of surgery

**Author and year**	**Country and study period**	**Definition of outcome**	**Reported risk estimates (95% CI)**^a^				**Key findings**
			**IBD**	**CD**	**UC**	**Estimate type**	
AbouKhalil 2018 [33]	Canada Jan/1998-Dec/2004 and Jan/2005-Dec/2011	Colectomy including total colectomy, total proctocolectomy with end ileostomy, or with ileal pouch anal anastomosis (IPAA)			1.47 [1.26;1.72]	HR	Male gender significantly more likely to have colectomy than women (1.47 [1.26;1.72]) No difference in risk of colectomy by economic deprivation in univariate analysis (Low deprivation (reference); Moderate 1.03 [0.83–1.29]; High 1.05 [0.84–1.31]
Chhay 2015 [35]	UK Jan/1989—Dec/2009	Colectomy (defined using Read or OXMIS coding including procedure codes for total colectomy and ileostomy, sub-total colectomy and proctocolectomy.)			1.44 [1.19;1.73]	HR	Men were more likely to undergo colectomy (HR 1.44, 95% CI: 1.19;1.73, P < 0.001) after adjusting for significant risk predictors (era of diagnosis, sex, age at diagnosis, thiopurine use, early steroid use and 5-ASA use)
Gao 2012 [39]	China Jan/2003—Dec/2010	Any type of intestinal resection except for merely stoma; abdominal or perianal abscess drainage and fistulectomy. The resection type was classified as small intestine resection; ileocolonic resection; and colectomy		1.99 [1.29;3.08]		HR	Male sex (HR, 1.99; 95% CI, 2.91;3.08; p = 0.002) was significantly associated with an increased risk for primary surgery
Gracie 2018 [41]	UK Sept/2014—Jun/2017	Intestinal resection	0.53 [0.15;1.85]			HR	Gender was not a predictor of intestinal resection
Kim 2017 [43]	South Korea Mar/1987—Dec/2013	Intestinal resection		0.84 [0.59;1.19]		HR	Gender was not associated with risk intestinal resection
Liu 2022 [70]	China Jan/2000 – Dec/2020	CD-related surgery		1.8 [1.07;3.01]		HR	Multivariate Cox regression analysis revealed that male sex (HR: 1.80, 95% CI: 1.07;3.01, p = 0.028) was an independent risk factor for surgery in the patients of A2 subtype (16 < age at diagnosis ≤ 40 years). Though the female patients had higher proportions of surgery following diagnosis both in A1 (age at diagnosis < 16) (22.0% vs. 25.0%) and A3 (age at diagnosis > 40) (24.0% vs. 26.0%) subtype, there were no significant differences, respectively
Nguyen 2023 [71]	USA 2010—2017	IBD-related surgery	1.13 [0.78;1.64]			HR	Sex was not associated with risk of IBD-related surgery within 1 year of biologics initiation
Peyrin-Biroulet 2012 [25]	USA Through March 2009	First major abdominal surgery		1,6 [1.02;2.4]		HR	Males significantly more likely to have surgery than females (p = 0.07)
Rinawi 2016 [49]	Israel 1981—2013	Intestinal surgery		0.98 [0.69;1.41]		HR	Sex was not a predictor for intestinal surgery (p = 0.924)
Rinawi 2017 [50]	Israel 1981- 2013	Colectomy			4.17 [1.75;10.00]	HR	Males significantly more likely to undergo surgery (p = 0.001)
Samuel 2013 [51]	USA 1970—2006	Any UC-related surgical hospitalization			2,1 [1.3;3.4]	HR	Males with UC were twice as likely as females to require colectomy (HR, 2.1; 95% CI, 1.3;3.4)
Sato 2015 [52]	Japan 1985—2010	Surgery defined as bowel resection or surgery for intestinal complications and did not include small operations for anal lesions or intraperitoneal drainage. Stenosis, fistula, and bleeding were the reasons for surgery		1.29 [0.98;1.70]		HR	Male sex had a borderline statistically significant association with risk of surgery when adjusted for age (1.02 (1.00–1.03), p < 0.05) but not when adjusted for age, smoking and alcohol drinking (1.29 (0.98–1.70))
Targownik 2012 [58]	Canada 1995—Mar/2008	Early colectomy (≤ 90 days from diagnosis date) and late colectomy (> 90 days from diagnosis date)			2.63 / 1.22 [1.58 / 0.97;4.36 / 1.53] Reported for early colectomy / late colectomy	HR	Colectomy rates were higher for men, both for early colectomy (males vs. females: 2.6% vs. 1.1%; hazard ratio (HR): 2.63, 95% CI: 1.58;4.36; P (univariate) = 0.0009) and for late colectomy (males vs. females: HR: 1.22, 0.97;1.53; P (univariate) = 0.036)
Targownik 2014 [59]	Canada 1987—Mar/2010	Resective surgery after the first year of disease	1,01 [0.83;1.24]			HR	No association between gender and risk of resective surgery was found
Zhao 2019 [63]	Denmark Jan 2003—Dec 2011	Surgery is defined in three different ways: Major surgery/Intestinal resection/Colectomy		0.98 / 2.5 / 0.78 [0.61 / 1.18 / 0.47;1.56 / 5.26 / 1.32] Reported for Major surgery/Intestinal resection/colectomy		HR	Female sex was statistically significantly associated with lower risk of undergoing intestinal resection. No associations between sex and major surgery or colectomy were found
Akintimehin 2018 [34]	Ireland Jan/2009—Dec/2025	Requiring a colectomy post 2011			0.91 [0.01;62.5]	OR	Gender did not show a statistical significance in predicting colectomy (OR: 1.1 [0.016–73.868], p-value: 0.965)
DeCristofaro 2022 [65]	Italy 2010—2020	Colectomy at any time during follow-up			0.18 [0.02;1.45]	OR	Sex was not significantly associated with odds of colectomy
King 2020 [44]	UK Apr/2017—Mar/2017	Risk of colectomy during emergency admission and colectomy by 12 months after emergency admission			1.37 / 1.35 [1.18 / 1.14;1.61 / 1.61] Colectomy during admission / Colectomy by 12 months	OR	A reduced risk of colectomy during the index admission and at 12 months was clearly observed in females (OR 0.73 [0.62;0.85], *p* < 0.001 and OR 0.74 [0.62;0.88], *p* < 0.001, respectively) Lower deprivation levels were associated with an increased risk of colectomy during index admission compared to the lowest deprivation level 1 (reference): Level 2: 1.46 [1.14–1.89], *p* = 0.003; Level 3: 1.35 [1.04–1.75], p = 0.023; Level 4: 1.60 [1.24–2.07], *p* = < 0.001; Level 5: 1.27 [0.97–1.66], *p* = 0.083 This was not observed at 12 months after admission: Level 2: 1.04 [0.78–1.37], *p* = 0.795; Level 3: 1.06 [0.80–1.40], *p* = 0.666; Level 4: 1.29 [0.98–1.7], *p* = 0.072; Level 5: 1.20 [0.91–1.59], *p* = 0.201
Lee 2023 [66]	USA Oct/2015—Dec/2019	Minimally invasive surgery	0.77 [0.71;0.87]			OR	Female sex was associated with higher odds of receiving minimally invasive surgery
Meregaglia 2015 [47]	Italy 2005 / 2008 / 2011	Gastrointestinal surgery if the DRG code was of a surgical type and categorised as belonging to the Major Diagnostic Category 6 Digestive System [MDC 6]		1.19 [1.14;1.25]	1.30 [1.20;1.39]	OR	Gender [OR = 0.84, female vs male; *p* < 0.001] was an independent factor affecting the risk of gastrointestinal surgery in Crohn's patients
Sceats 2019 [53]	USA 2008—2014	Any index ulcerative colitis operation			1.51 [1.32;1.73]	OR	After adjusting for covariates, including age group, geographic region, insurance plan type, and grouped Charlson comorbidity index score, men had higher risk of surgery (OR = 1.51, 95% CI = [1.32;1.73], *p* < 0.001)
Stamatiou 2022 [67]	UK 2000—2020	Requirement for intra-abdominal surgery within 5 years of first contact with the surgical services		0.63 [0.45;0.88]	1.23 [0.74;2.06]	OR	Females with CD may be more likely to undergo surgery than males with CD (*p* = 0.008)
Sun 2019 [56]	China Jan/2013—Dec/2018	Abdominal surgery within the first year after CD diagnosis		4.67 [2.68;8.13]		OR	Female sex was associated with a statistically significantly lower risk of abdominal surgery within the first year after CD diagnosis
Tanaka 2021 [57]	Japan Aug/2008—Mar/2020	Colectomy			0.857 [0.261;2.815]	OR	Sex was not associated with risk of colectomy
Wan 2023 [68]	China Mar/2019 – Jan/2023	Any IBD-related surgery involving bowel resection		1.04 [0.86;1.24]	0.84 [0.51;1.37]	OR	No difference in surgery rates between men and women
Wang 2022 [69]	China 2016 – Nov/2021	Surgical treatment		1.41 [1.02;1.95]	6,99 [0.85;55.56]	OR	Female sex is a predictor of lower risk of surgical treatment for CD patients, but not UC patients
Wong 2019 [62]	USA 2007—2015	Binary measure of any IBD-related surgery measured by CPT codes: 43,020–43135, 43,280–44346, 44,500–45190, 45,395–46,505, 46,700–46999, and 49,000–49999	1.25 [1.11;1.25] / 1.43 [1.11;1.67] Reported for Total population / Pediatric population			OR	Female sex was associated with a lower risk of undergoing IBD-related surgery in total population and in the pediatric population
Eder 2017 [37]	Poland Jan/2014—Dec/2015	Intestinal resection		Males: 18.2 Females: 23.0 *p-value* = 0.34		%	No difference in surgery rate between males and females (*p* = 0.340)
Goel 2013 [40]	India Jan/1995—Dec/2008	Need for surgery		Males: 74.0 *p*-value = 0.01		%	Male sex was a significant predictor of surgery (adjusted OR 2.9; *p* = 0.008)
Winder 2019 [61]	Israel 2006—2014	Bowel resection surgery and stricture surgery at follow-up		Males: 42.9 *p*-value = 0.80		%	No difference between males and females for stricture surgery per patient at follow up for Endoscopic Balloon Dilation
Dotson 2015 [36]	USA Apr/2004—Jun/2012	Colectomy		Males: 225 (8) Females: 211 (7) *p*-value = 1		N(%)	No sex difference was observed for colectomy, abdominal surgery or other surgery modalities
Heath 2022 [64]	Canada Mar/2012—Sept/2019	Surgery: Any surgery and Multiple surgeries		Males: 106 (39.4) Females: 116 (30) *p*-value = 0.02	Males: 41 (15.2) Females: 42 (10.9) *p*-value = ns	N (%)	Males with CD are significantly more likely than females with CD to have any type of surgery (39.4% vs 30%, *p* = 0.015)
Herzog 2014 [42]	Switzerland 2008—Sep/2012	Colectomy		2 (11.1) / 8 (16.3) p-values = 0.83 / 0.73 Reported for the age groups < 10 years at diagnosis / ≥ 10 years at diagnosis	3 (17.7) / 0 *p*-value = 0.30 Reported for the age groups < 10 years at diagnosis / ≥ 10 years at diagnosis	N (%)	Gender was not associated with frequency of colectomy in CD or UC patients
Lie 2017 [45]	Netherlands Mar/2006—Feb/2011	Intestinal surgery		Males: 31 (38.3) Females: 42 (39.3) *p*-value = 0.89		N (%)	Gender was not statistically significantly associated with previous intestinal surgery (*p* = 0.891) at start of adalimumab treatment
Magro 2019 [46]	Portugal 2015—2016	Need to undergo a colectomy			Males: 36 (7) Females: 33 (5) *p*-value = 0.19	N (%)	No difference in colectomy rate between males and females
Severs 2018 [26]	The Netherlands 2010—?	Small bowel resection / Ileocaecal resection / Colon resection		Males: 127 (16%) / 308 (40%) / 124 (16%) *p*-value = < 0.01 / < 0.01 / 0.22 Reported for Small bowel resection / Ileocaecal resection / Colon resection	Males: 102 (17%) *p*-value = 0.54 Reported for colon resection	N(%)	1. In the Dutch IBD Biobank, more male CD patients underwent small bowel and ileocecal resection than female CD patients (16% versus 9%, and 40% versus 33%, respectively, both *P* < = 0.01). No further differences in surgery rates between men and women (colon resection, ileostomy, colostomy, abscess/fistula surgery, and pouches) were observed. These observations were confirmed in another cohort (the COIN study)
Solberg 2015 [54]	Norway 1990—1994	Colectomy during the 10 years of follow-up			p-value = 0.51	N (%)	Sex was not a predictive factor for colectomy
Gajendran 2016 [38]	USA 2009—2011	Major surgical intervention during the hospitalization after an emergency department visit	1.04 [0.99;1.10]			OR	No association between gender and major surgical intervention No association between lowest income group compared with highest income group (OR: 1.04 [0.97;1.12], p = 0.275)
Stokes 2018 [55]	USA 2003, 2006, 2009, 2012	Intestinal resection (any type)		1.41 [1.28;1.56]		OR	Female patients had lower odds of undergoing resection compared to men (odds ratio [OR] 0.71, 95% confidence interval [CI] 0.64 to 0.78)
Timmer 2017 [60]	Germany 2011/2012 and 2013	Resecting surgery (ever)		0.74 [0.50;1.09]		OR	Sex: No difference in surgery rate between males and females; SES: no difference in surgery rate between the middle income tertile and the high (1.27 [0.76;2.13] and low tertile (0.85 [0.53;1.38], respectively
Bernstein 2020 [72]	Canada Apr/1995—Mar/2018	Surgery for IBD identified by physician billing codes for intestinal suture, colostomy, colectomy, proctectomy, proctosigmoidectomy, small bowel resection or ileostomy, or hospital procedure codes for incision, excision, and anastomosis of intestine (excluding diagnostic procedures)					Those with lower socioeconomic status were more likely to have surgery compared to those not having lower socioeconomic status (HR = 1.11 [1.00;1.23])
Li 2015 [73]	China Sep/2010 – Aug/2014	Intestinal surgery including resections, colectomy, ileostomy, colostomy and appendicectomy					No difference in surgery between basic and higher education (OR: 0.802 [95% CI: 0.467;1.379], *p* = 0.426)
McLoughlin 2020 [74]	USA 2006–2012	Major surgical procedure / total abdominal colectomy / small bowel resection / large bowel resection					1st quartile of parental income was associated with lower risk of major surgical procedure and small bowel resection compared to 2nd, 3rd and 4th income quartiles (Q2: 1.16 (1.03–1.30) *p* = 0.02 / 1.56 (1.15–2.11) *p* = 0.01 / Q3: 1.22 (1.08–1.37) *p* < 0.01 / 1.53 (1.14–2.06) *p* = 0.01 / Q4: 1.28 (1.12–1.45) *p* < 0.01 / 1.68 (1.25–2.26) *p* < 0.01). No significant associations between parental income and total abdominal colectomy and large bowel resection were found
Osamura 2018 [48]	Japan Patients treated before Oct/2014	Risk of surgery					No sex differences for surgery (results not reported)

^a^all risk estimates reported for males compared with females. Risk estimates for socioeconomic variables are reported in key findings

**Fig. 2:**
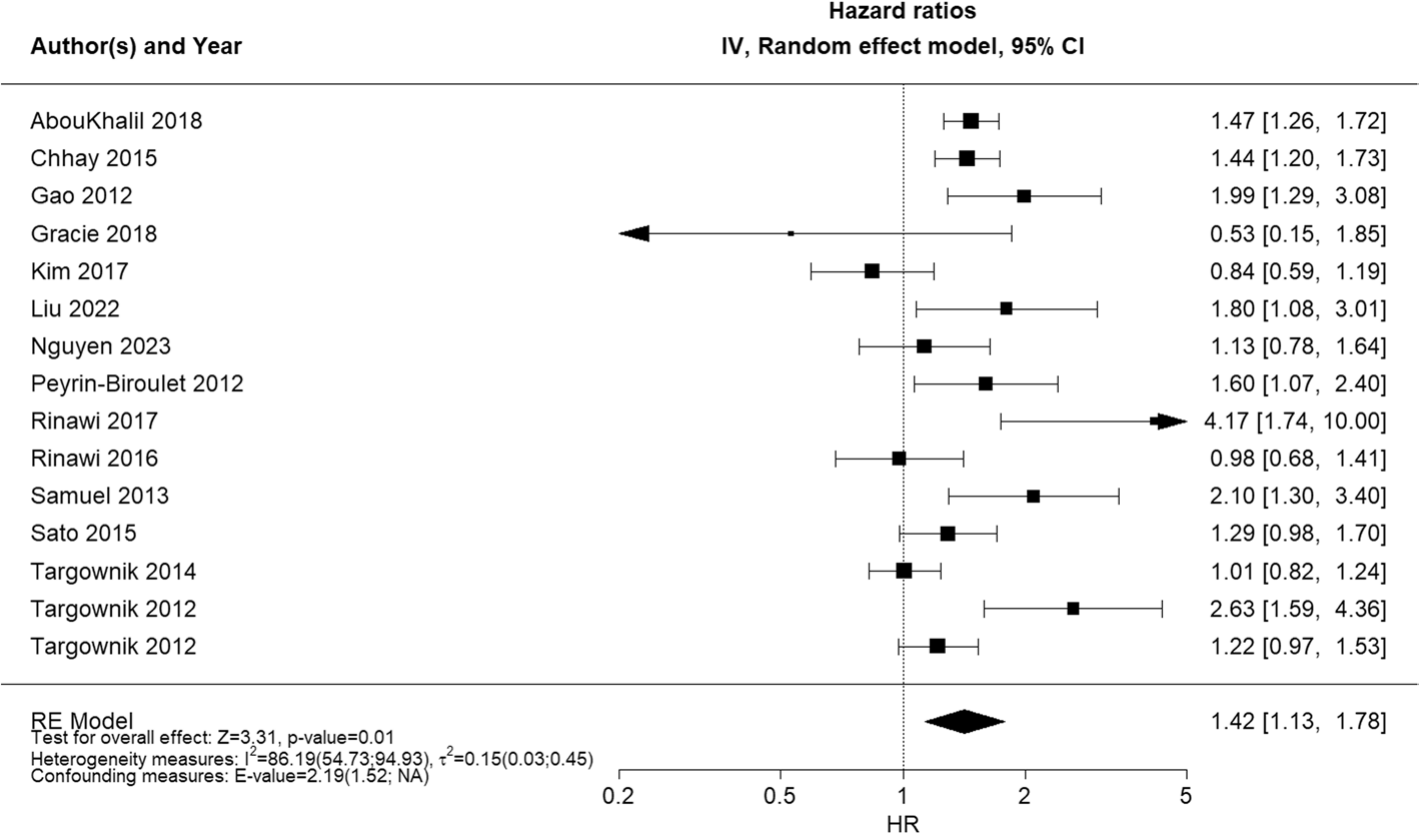
Forest plot of RE model meta-analysis of HRs for the likelihood of surgery across included studies Forest plot of RE model meta-analysis of HRs for the likelihood of surgery in male patients compared to female patients with IBD across included studies. Reference: female patients. Abbreviations: HR, hazard ratio; IBD, inflammatory bowel disease; NA, not applicable; RE, random effects

The effect estimates of studies on sex differences eligible for meta-analysis (14 studies for surgery) were pooled to present an overall HR of 1.42 (95% CI 1.13; 1.78, Fig. 2), indicating a higher rate/probability of surgery among male patients than female patients. The heterogeneity measures for surgery showed an *I*^*2*^ = 86.19 [54.73; 94.93]% and ${{\varvec{\tau}}}^{2}$ = 0.15 [0.03; 0.45], which suggests substantial heterogeneity between the studies included.

A total of six studies (9%) compared bowel surgery across income groups [33, 38, 44, 60, 72, 74], and three of these (50%) identified statistically significant income-based differences, with two of the three studies indicating that individuals with a lower income had a lower likelihood of bowel surgery [44, 74]. Conversely, only two studies (3%) examined education level and the likelihood of surgery and found no statistically significant associations [60, 73].

**Fig. 3:**
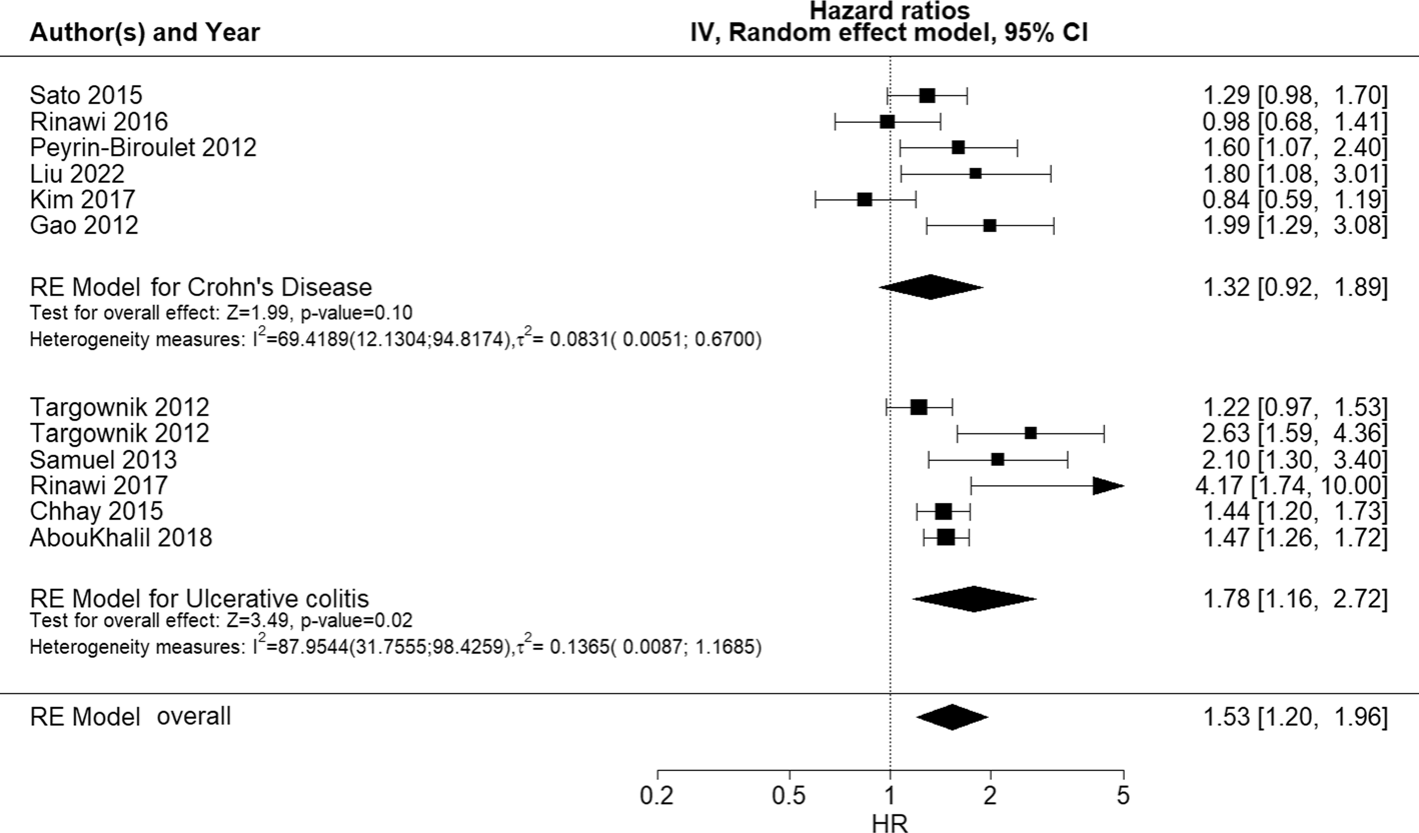
Forest plot of subgroup (CD/UC) meta-analysis of HRs for risk of surgery Forest plot of RE model subgroup meta-analysis of HRs for risk of surgery in male patients compared to female patients with IBD by IBD subtype. Reference: female patients. Abbreviations: HR, hazard ratio; IBD, inflammatory bowel disease; NA, not applicable; RE, random effects

In subgroup analyses, the pooled estimate for the likelihood of surgery in males compared to females was statistically significant for studies of UC (HR 1.78 [95% CI 1.16;2.72]), whereas not for studies of CD (HR 1.32 [95% CI 0.92;1.89]), with a higher likelihood among males than females (Fig. 3). The estimate for *I*^*2*^ in the CD analysis is wider but has consistently lower values for both *I*^*2*^ and ${{\varvec{\tau}}}^{2}$ (CD (*I*^*2*^ = 69.42 [12.13;94.82]% and ${{\varvec{\tau}}}^{2}$ = 0.08 [0.005;0.67]) versus UC: (*I*^*2*^ = 87.95 [31.76;98.43]% and ${{\varvec{\tau}}}^{2}$ = 0.14 [0.009;1.17]). Thus, the IBD type may explain some of the between-study heterogeneity in the main RE model.

**Fig. 4:**
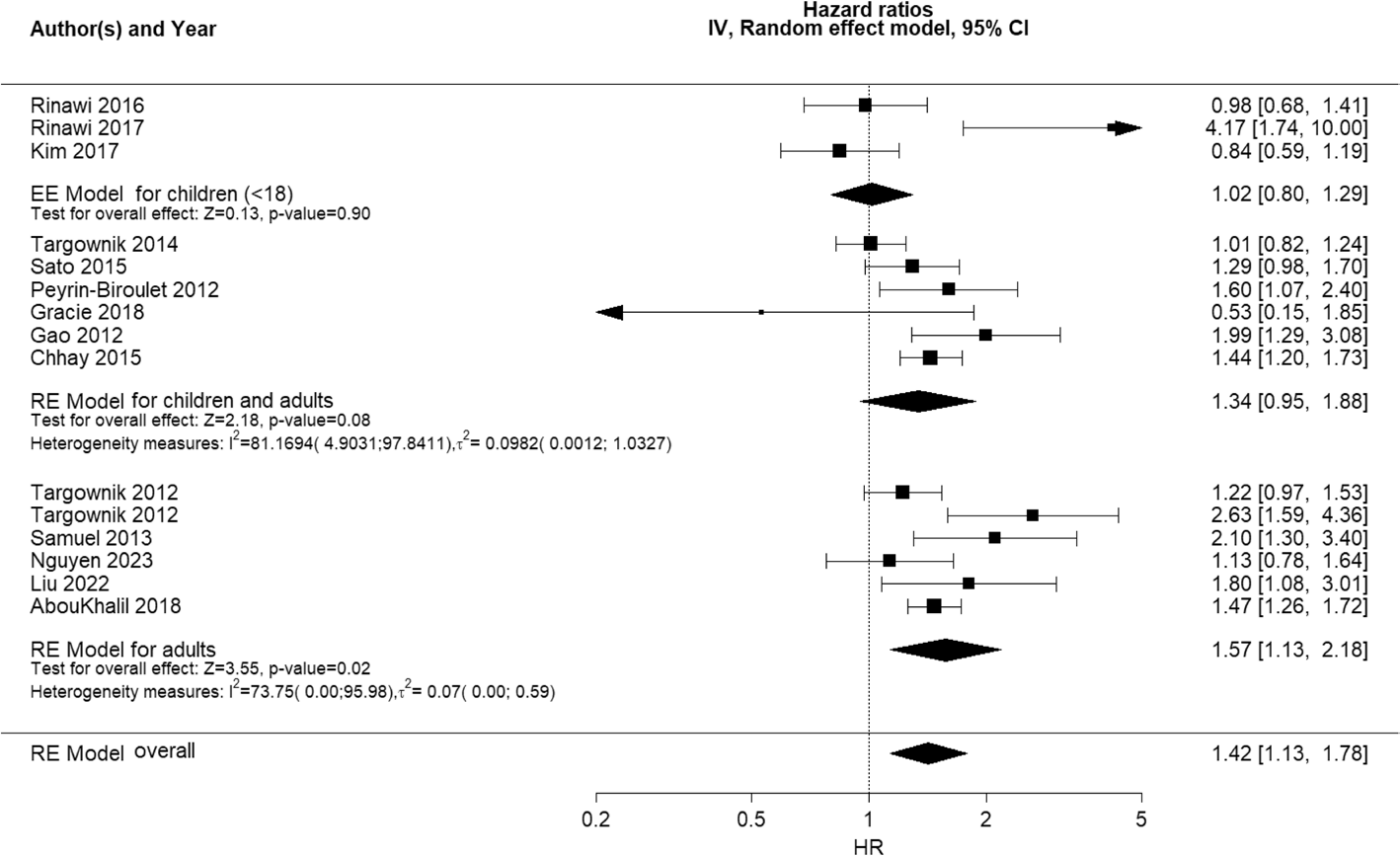
Forest plot of subgroup (children/adults) meta-analysis of HRs for risk of surgery Forest plot of RE model subgroup meta-analysis of HRs for risk of surgery in male patients compared with female patients with IBD by children and adults subgroups. Reference: female patients. Abbreviations: HR, hazard ratio; IBD, inflammatory bowel disease; NA, not applicable; RE, random effects

When stratifying the studies of surgery by age, a statistically significant sex difference was found for studies of adults (HR 1.57 [95% CI 1.13;2.18]), but not for those of children (HR 1.02 [95% CI 0.80;1.29]), with a higher likelihood of surgery among adult males compared to adult females (Fig. 4). A fixed effects model was undertaken for studies of children instead of a random effects model for adults, as the between-study heterogeneity was too low to be estimated sufficiently, with only three studies focusing on children separately. For the adult model, the overall study heterogeneity was also low (${{\varvec{\tau}}}^{2}$ = 0.07 [0.00;0.59]). Based on the low heterogeneity measures, stratification by children/adults may explain some of the between-study heterogeneity in the main RE model.

**Fig. 5:**
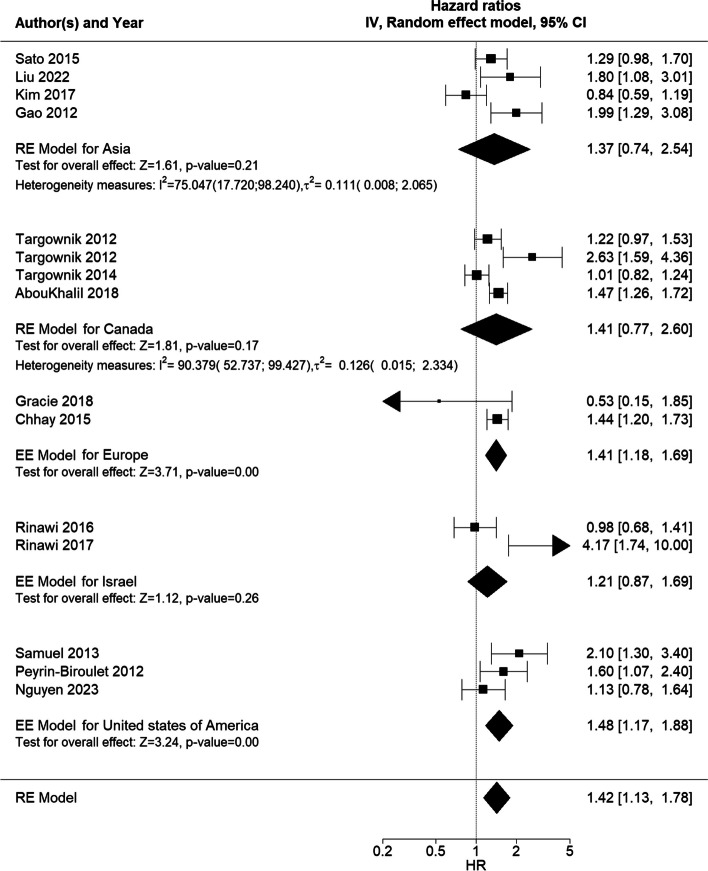
Forest plot of subgroup (country) meta-analysis of HRs for risk of surgery Forest plot of RE model subgroup meta-analysis of HRs for risk of surgery in male patients compared with female patients with IBD by country. Reference: female patients. Abbreviations: HR, hazard ratio; IBD, inflammatory bowel disease; NA, not applicable; RE, random effects

Further subgroup analyses by country revealed that the country where the study was conducted might also explain some of the between-study heterogeneity in the main RE model (Fig. 5).

### Hospitalization

In 17 studies (25.4%) comparing the likelihood of hospitalization by sex [26, 38, 41, 48, 51, 64, 71, 75–84], male patients were significantly more likely to be hospitalized in 8 studies [38, 48, 75, 76, 81–84], female patients were significantly more likely to be hospitalized in 2 studies [64, 80], and there was no significant difference in 7 studies [26, 41, 51, 71, 77–79] (Table 2).

**Table 2:** Reported relative risk estimates and key findings in studies of hospitalization

**Author and year**	**Country and study period**	**Definition of outcome**	**Reported relative risk estimates (95% CI)**^a^				**Key findings**
			**IBD**	**CD**	**UC**	**Estimate type**	
Barnes 2017 [76]	USA 2013	Hospital readmission within 90 days of the index hospitalization		1.36 [1.03;1.81]	0.81 [0.58;1.14]	OR	For CD: Male sex (aOR 1.36, 95% CI 1.03–1.81) were associated with a significant increase in odds of readmission within 90 days of index hospitalization For UC: no significant sex difference (aOR 0.81 95% CI 0.58–1.14)
Chudy-Onwugaje 2021 [77]	USA Nov/2012—Dec/2015	Total number of IBD-related hospitalizations in the 12-month period after a patient's first visit to the practice. High utilization group = 2 or more hospital admissions. Low utilization group = no visits	1.12 [0.75;1.69]	1.30 [0.79;2.08]	0.89 [0.40;2.00]	OR	No difference in health care utilization between males and females
Gajendran 2016 [38]	USA 2009—2011	Hospitalization after an emergency department visit	1.15 [1.12;1.16]			OR	Male gender (OR 1.17) was associated with higher odds of hospitalization following an ED visit
Limsrivilai 2017 [79]	USA Jul/2012—Jun/2015	Number of IBD-related hospitalizations	0.8 *p*-value = 0.15			OR	Gender was not a predictor of IBD-related hospitalization
Mandel 2014 [80]	Hungary Jan/2008—?	All-cause and IBD-related hospitalizations (including medical/surgical complications) before and during anti-TNF therapy		0.52 [0.26;0.97]	ns	OR	In CD, the risk of hospitalization in the two years prior to anti-TNF therapy was associated with female gender (*p* = 0.04,OR:1.92, 95%CI:1.03;3.82). The risk of hospitalization during anti-TNF therapy was not associated with gender in neither uni- or multivariate analyses (*p* = 0.43 for CD) In UC, gender was not associated with the need for hospitalization
Micic 2017 [81]	USA ?—2013	All-cause 30-day readmission from discharge date of index hospitalization	1.16 [1.07;1.25]			OR	Male sex (OR: 1.16; 95% CI 1.07–1.25) was associated with an increased risk of readmission Higher quartiles of household income (> $64,000; OR: 0.88; 95% CI 0.79–0.98) were associated with a decreased rate of readmission when compared to the lowest quartile of income($1-$37,999)
Mudireddy 2017 [82]	USA Jan/2007—Dec/2010	Readmission within 1 month, 3 months or 1 year from the index admission	1.58 [1.05;2.37]			OR	Within 1 year hospital readmission was statistically significantly associated with being male (OR: 1.58; 95% CI, 1.05;2.37; *P* = 0.03). Within 1 months or 3 months hospital readmissions were not statistically significantly associated with gender
Osamura 2018 [48]	Japan Patients treated before Oct/2014	Risk of hospitalization 1 year, 5 years and 10 years after initiation of anti-TNF therapy		1.43 *p*-value = 0.044		OR	Male sex (OR = 0.70, *P* = 0.044) was a risk factor for hospitalization
Poojary 2017 [83]	USA 2013	30-day readmission			1.30 [1.12;1.52]	OR	Patients were less likely to be readmitted if they were women (0.77 (0.66–0.89), *p* = 0.0003) No statistically significant association between household income and risk of 30-day readmission was found
Reja 2020 [84]	USA Jan/2016—Dec/2016	Readmission for opioid dependence, which was defined as readmission with a principal or secondary diagnosis of opioid dependence after an index admission free of this diagnosis	0.66 [0.52;0.84]	1.64 [1.06;2.56]	0.58 [0.52;1.12]	OR	For total IBD, female sex was an independent predictor of increased risk of readmission (aOR 1.51, 95% CI 1.19;1.92;*p* < 0.05) For CD, female sex was an independent predictor of decreased risk of readmission for CD (0.61 (0.39;0.94), *p* < 0.05) For UC, no significant association
Gracie 2018 [41]	UK Sept/2014—Jun/2017	Hospitalization due to Disease Activity	1.18 [0.60;2.33]			HR	Gender was not a predictor of hospitalization
Nguyen 2023 [71]	USA 2010—2017	All-cause hospitalization	1.07 [0.93;1.22] *p*-value = 0.36			HR	Sex was not associated with risk of all-cause hospitalization within 1 year of biologics initiation
Samuel 2013 [51]	USA 1970—2006	Any UC-related hospitalization			1.1 [0.8;1.6]	HR	No statistically significant difference between males and females for risk of hospitalization
Gunnells 2015 [78]	USA 2012—2013	All-cause readmission within 30 days of index surgery	Males: 187 (49) Females: 192 (51) *p*-value = 0.48			N (%)	No difference in readmission rate between males and females
Heath 2022 [64]	Canada Mar/2012—Sept/2019	Hospitalization		Males: 118 (43.9) Females: 190 (49.1) *p*-value = ns	Males: 74 (39.6) Females: 95 (55.2) *p*-value = 0.003	N (%)	Females with UC are significantly more likely than males with UC to be hospitalized (55.2% vs 39.6%, *p* = 0.003)
Severs 2018 [26]	The Netherlands 2010—?	Hospitalization due to IBD		Males: 19 (5%) Females: 17 (3%) *p*-value = 0.09	Males: 14 (3%) Females: 18 (4%) *p*-value = 0.28	N (%)	No differences in risk of hospitalization due to IBD were observed
Axelrad 2019 [75]	USA Jan/2007—Jun/2017	Inpatient hospitalization				IRR	Female sex was statistically significantly associated with a lower risk of inpatient hospitalization (IRR = 0.76 (0.69–0.84))
Bernstein 2020 [72]	Canada Apr/1995—Mar/2018	Hospitalization				HR	Those with lower socioeconomic status was more likely to be hospitalized (HR = 1.23 [1.13;1.34], *p*-values not reported

^a^all risk estimates reported for males compared with females. Risk estimates for socioeconomic variables are reported in key findings

**Fig. 6:**
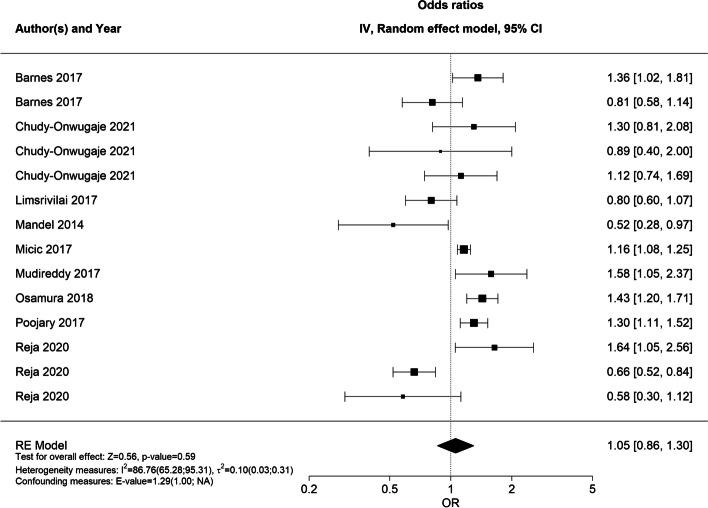
Forest plot of RE model meta-analysis of ORs for the likelihood of hospitalization across included studies Forest plot of RE model meta-analysis of ORs for the likelihood of hospitalization in male patients compared to female patients with IBD across included studies. Reference: female patients. Abbreviations: OR, odds ratio; IBD, inflammatory bowel disease; NA, not applicable; RE, random effects

In meta-analysis, an overall OR of 1.05 (95% CI 0.86;1.30, Fig. 6) was estimated for the 9 eligible studies, indicating no statistically significant sex difference in the rate/probability of hospitalization. The heterogeneity measures showed an *I*^*2*^ = 86.76 [65.28;95.31]% and ${{\varvec{\tau}}}^{2}$ = 0.10 [0.03;0.31], indicating substantial heterogeneity between the studies.

Three studies (4.5%) compared hospitalization across income groups [72, 81, 83], of which two studies (66.7%) identified a statistically significant association between high income and a lower likelihood of hospitalization [72, 81]. No studies examining the association between educational level and likelihood of hospitalization were identified.

**Fig. 7:**
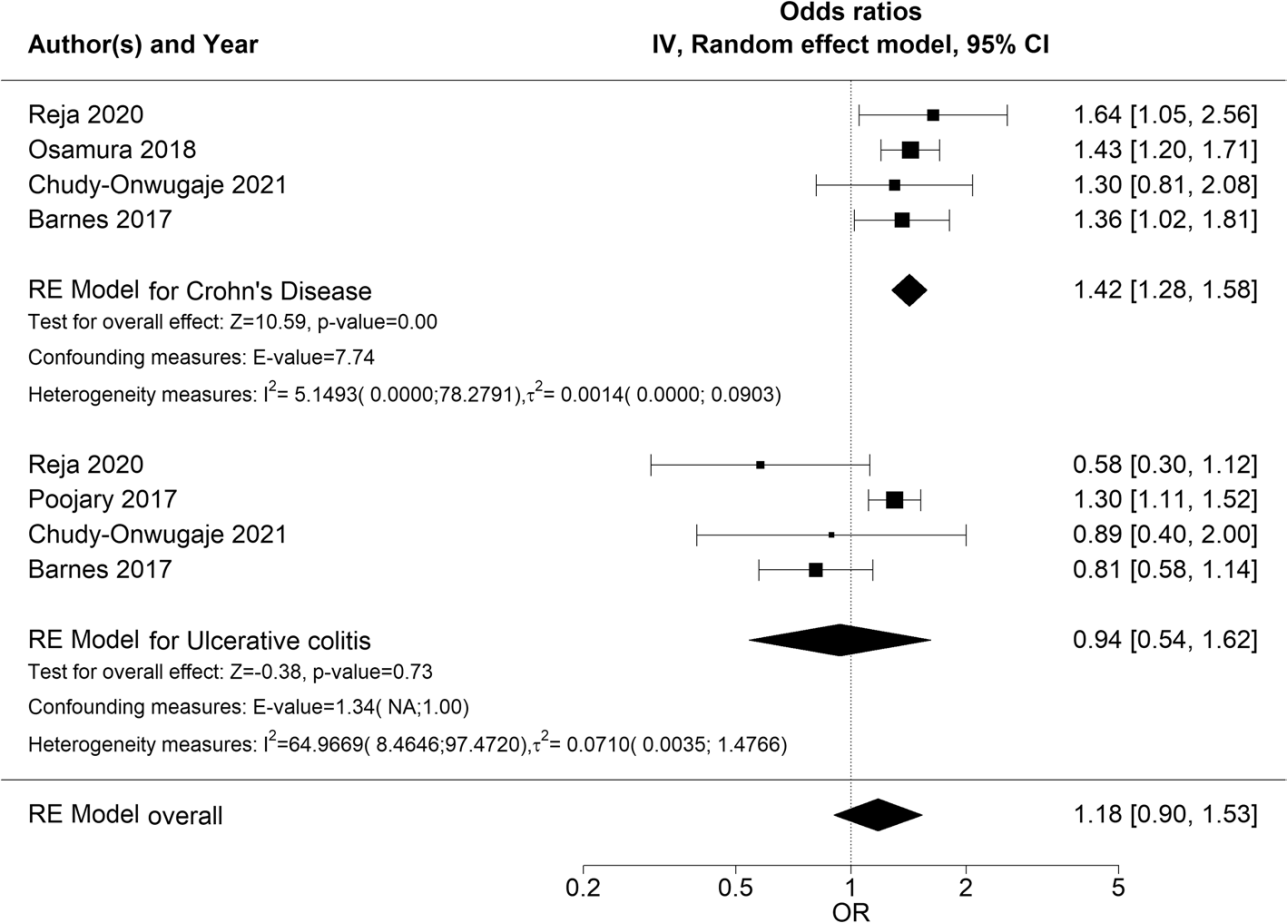
Forest plot of subgroup (CD/UC) meta-analysis of ORs for risk of hospitalization Forest plot of RE model subgroup meta-analysis of ORs for risk of hospitalization in male patients compared to female patients with IBD by IBD subtype. Reference: female patients. Abbreviations: OR, odds ratio; IBD, inflammatory bowel disease; NA, not applicable; RE, random effects

In the subgroup analyses, the male sex was significantly associated with a higher likelihood of hospitalization among patients with CD (OR 1.41 [95% CI 1.08;1.84]), but not UC (OR 0.94 [95% CI 0.54;1.62]) (Fig. 7). The overall heterogeneity in both RE models was very low (*I*^*2*^ = 5.97 [0.00;93.11]% and ${{\varvec{\tau}}}^{2}$ = 0.003 [0.000;0.55] and *I*^*2*^ = 5.97 [0.00;93.11]% and ${{\varvec{\tau}}}^{2}$ = 0.003 [0.000;0.55], respectively). Subgroup analysis on children/adults and country was not undertaken because of the low number of studies in the children subgroup (two), and the fact that most studies were from the USA (seven of nine studies).

### Use of corticosteroids

A total of 13 studies (19.4%) examined corticosteroid use [36, 42, 45, 59, 64, 70, 72, 75, 85–89] (Table 3), of which only 2 studies (15.4%) reported a statistically significant sex-based difference in the use of corticosteroids, with fewer male patients using a corticosteroid (budesonide) than female patients for both CD and UC in one study [64] and fewer female patients using steroids for total IBD patients in the other study [89].

**Table 3:** Reported relative risk estimates and key findings in studies of corticosteroids

**Author and year**	**Country and study period**	**Definition of outcome**	**Reported relative risk estimates (95% CI)**^a^				**Key findings**
			**IBD**	**CD**	**UC**	**Estimate type**	
Targownik 2014 [59]	Canada 1987—Mar/2010	Corticosteroid exposure in the first year and first 5 years of disease			1.04 [0.96;1.12] / 1.09 [0.99;1.19] Reported for 1 year / 5 years	HR	There was a trend toward higher corticosteroid exposure among males in the first year and first 5 years of disease, but not statistically significant
Heath	Canada Mar/2012—Sept/2019	Corticosteroids drug exposure		Males: 49 (18.4) Females: 110 (28.5) *p*-value = 0.003	Males: 12 (6.2) Females: 22 (12.7) *p*-value = 0.047	N (%)	Females with CD are significantly more likely than males to have been exposed to budesonide (corticosteroid)
Lie 2017 [45]	Netherlands Mar/2006—Feb/2011	Use of corticosteroids		Males: 39 (48.1) Females: 45 (42.1) *p*-value = 0.405		N (%)	Gender was not statistically significantly associated with use of corticosteroids (*p* = 0.405) at start of adalimumab treatment
Liu 2022 [70]	China Jan/2000 – Dec/2020	Use of glucocoticosteroids		Males: 310 (73.8) Females: 138 (72.3) *p*-value = 0.587		N (%)	No difference was observed in use of glucocorticosteroids at diagnosis between female and male patients
Dotson 2015 [36]	USA Apr/2004—Jun/2012	Use of corticosteroids		Males: 1798 (62%) Females: 1781 (62%) *p*-value = 1		N (%)	Female rates for corticosteroids (*n* = 1781, 62%) were not statistically different from those for males (*n* = 1798, 62%,*P* = 1) [adjusted based on Bonferroni correction]
Herzog 2014 [42]	Switzerland 2008—Sep/2012	Use of corticosteroids		Males: 14 (77.8) / 42 (85.7) Females: 5 (71.4) / 22 (71) *p*-value = 0.65 / 0.13 Reported for < 10 years / > = 10 years	Males: 17(100) / 16(70) Females: 21(84) / 20(76.9) *p*-value = 0.13 / 0.88 Reported for < 10 years / > = 10 years	N (%)	Gender was not associated with use of corticosteroids
McKenna 2018 [87]	USA Jan/2002—Aug/2013	Preoperative use of steroids		Males: 194 (55.0%) Females: 128 (58.7%) *p*-value = 0.38		N (%)	Gender was not associated with preoperative use of steroids
Sundel 2024 [89]	USA Mar/2017 – Mar/2021	Steroid use (yes/no)		Males: 997 (62.9) Females: 895 (58.1) *p*-value = 0.005		N (%)	Males significantly more likely to use steroids than females (62.9% vs 58.1%, *p* = 0.005)
Barkan 2024 [88]	Israel -	Treatment with corticosteroids in previous 12 months	1.64 [0.40;6.67]			OR	Sex was not associated with risk of steroid use
Lee 2012 [86]	USA May/2007—May/2010	Medication use with corticosteroids (Prednisone)		0.79 [0.49;1.27]	1.09 [0.60;1.96]	OR	No sex differences in the use of prednisone in the study population as a whole or when stratified by age
daSilva 2015 [85]	Brazil Jan/2011—Sep/2012	Use of systemic corticosteroids therapy			0.82	Prevalence ratio	Gender was not statistically significantly associated with use of corticosteroids
Axelrad 2019 [75]	USA Jan/2007—Jun/2017	Ever requiring steroids	%1.%2 [0.75;1.33] *p*-value = 0.98			Rate ratio	Sex was not statistically significantly associated with use of steroids
Bernstein 2020 [72]	Canada Apr/1995—Mar/2018	Use of cortiocosteroids					Those with lower socioeconomic status was more likely to take corticosteroids (HR = 1.12 [1.03;1.21]

^a^all risk estimates reported for males compared with females. Risk estimates for socioeconomic variables are reported in key findings

No studies examining adherence to corticosteroids were found.

One study (1.5%) compared corticosteroid use across income groups and concluded that those with lower income were more likely to take corticosteroids [72]. However, no studies on educational level and use of corticosteroids were found.

### Use of biological therapy

Twelve studies (17.9%) examined the use of biologics [26, 36, 42, 60, 64, 70, 72, 86, 87, 90–92] (Table 4). Only 2 studies found statistically significant sex differences, with female CD patients having a lower probability of using biologics than male patients in one study [64], and a similar association, but for UC patients only, in the other study [92].

**Table 4:** Reported relative risk estimates and key findings in studies of biological treatment

**Author and year**	**Country and study period**	**Definition of outcome**	**Reported relative risk estimates (95% CI)**^a^				**Key findings**
			**IBD**	**CD**	**UC**	**Estimate type**	
Lie 2017 [45]	Netherlands Mar/2006—Feb/2011	Discontinuation		1.81 *p*-value = 0.02		HR	Males significantly more likely to continue ADA treatment (HR = 1.807, *P* = 0.020)
Rundquist 2018 [93]	Sweden 2005—2017	Discontinuation of golimumab treatment		0.15 [0.02;0.96] / 0.45 [0.24;0.84] Reported for at 12 weeks / at most recent foloow-up		HR	Male sex was associated with decreased risk of discontinuing golimumab treatment at 12 weeks adjusted HR = 0.15, 95% CI: 0.02;0.96;*p* = 0.05) and at the most recent follow-up (adjusted HR = 0.45, 95% CI: 0.24;0.84;*p* = 0.01)
Schultheiss 2019 [94]	The Netherlands Jan/2011—Dec/2017	All TNF-alfa inhibitor drug persistence	0.70 [0.57;0.86]			HR	Male sex was significantly associated with a lower risk of discontinuation of TNF-alfa inhibitor therapy in multivariate Cox proportional hazards regression (HR 0.70, 95% CI 0.57;0.86)
Tanaka 2018 [95]	Japan Between Oct/2010 and Dec/2013	Retention of adalimumab treatment defined as the incidence of the discontinuation of adalimumab treatment		0.73 [0.56;0.94]		HR	Female sex was identified as independent predictor for the discontinuation of adalimumab
Heath	Canada Mar/2012—Sept/2019	Biologics drug exposure		Males: 11 (3.8%) Females: 18 (4.5%) *p*-value = ns	Males: 28 (14.8%) Females: 14 (8.0%) *p*-value = 0.049	N (%)	Females with UC are significantly more likely than males to have been exposed to biologics
Herzog 2014 [42]	Switzerland 2008—Sep/2012	Anti-TNF therapy		Males: 6 (33.3) / 11(22.5) Females: 4 (57.1) / 13 (41.9) *p*-value = 0.20 / 0.14 Reported for < 10 years / > = 10 years	Males: 6 (35.3) / 4 (14.4) Females: 3 (12) / 1 (3.8) *p*-value = 0.45 / 0.17 Reported for < 10 years / > = 10 years	N (%)	Gender was not associated with use of anti-TNF therapy
Lagana 2019 [96]	Italy Before 2019	Discontinuation of adalimumab or inflimax	Males: 8 (9%) / 30 (27.5%) Females: 17 (22%) / 25 (32%) *p*-value = 0.03 / 0.52 Reported for adalimumab / infliximab			N (%)	The overall rate of female patients discontinuing ADA (17/77, 22%) was significantly (*p* = 0.03) higher than that of male patients (8/85, 9%) No significant differences between female and male patients were detected for IFX discontinuation
Liu 2022 [70]	China Jan/2000 – Dec/2020	Use of biologic therapy		Males: 122 (29.0) Females: 47 (24.6) *p*-value = 0.255		N (%)	No difference was observed in use of biologics at diagnosis between female and male patients
Severs 2018 [26]	The Netherlands, 2010—?	Anti-TNF, Adalimumab, Infliximab		Males: 81 (22%) / 35 (10%) / 47 (13%) Females: 118 (21%) / 67 (12%) / 51 (9%) *p*-value = 0.63 / 0.28 / 0.06 Reported for Anti-TNF/Adalimumab/Infliximab	Males: 18 (3%) / 8 (2%) / 10 (2%) Females: 21 (4%) / 5 (1%) 16 / (3%) *p*-value = 0.37 / 0.54 / 0.13 Reported for Anti-TNF/Adalimumab/Infliximab	N (%)	No differences regarding the use of biologics were observed between men and women
Dotson 2015 [36]	USA Apr/2004—Jun/2012	Use of biological agent		Males: 444 (15%) Females: 460 (16%) *p*-value = 1		N(%)	Female rates for biological agents (*n* = 460, 16%), were not statistically different from those for males (*n* = 444, 15%,*P* = 1) [adjusted based on Bonferroni correction]
Khalili 2020 [90]	Sweden Jan/2014—Dec/2014	Anti-TNF treatment	Females: 1016 (42.5%)	Females: 674 (44%)	Females: 342 (39.7%)	N (%)	No description
McKenna 2018 [87]	USA Jan/2002—Aug/2013	Preoperative use of biologics		Males: 71 (20.2%) Females: 48 (22.1%) *p*-value = 0.58		N(%)	Gender was not associated with preoperative use of biologics
Calvo-Arbeloa 2020 [97]	Spain Jan/2019—Jun/2019	Adherence to treatment with adalimumab, golimumab and ustekinumab	2.28 [1.13;4.63]			OR	Female sex was associated with lower adherence levels/male patients had higher odds for being adherent to biologics; No statistical significant association between educational status and adherence was found
Lee 2012 [86]	USA May/2007—May/2010	Medication use with biologics therapy (Infliximab)		0.96 [0.72;1.27]	0.71 [0.38;1.30]	OR	No sex differences in the use of infliximab in the study population as a whole or when stratified by age
Lin 2013 [91]	USA 1998—2010	Use of anti-TNF therapy	0.85 [0.35;2.08]			OR	Gender was not associated with anti-TNF therapy
Mahlich 2018 [92]	Japan Survey data collected in Feb/2016	Biological treatment not further specified	1.30 [0.78;2.17]	3.33 [1.27;9.09]	1.49 [0.28;1.30]	OR	In Crohn’s disease, female IBD patients had lower probability of using biologic agents than men. No statistically significant associations were found in UC patients Annual income did not play a role in selecting biologic treatment in Japan The highest level of education (masters degree or higher) was associated with lower risk of biological treatment compared to the lowest education (college or less) in Crohns disease patients and overall
Timmer 2017 [60]	Germany 2011/2012 and 2013	Use of biologics (ever)	0.83 [0.62;1.10]			OR	Sex: No difference in use of biologicals between males and females; SES: no difference in use of biologicals between the middle income tertile and the high and low tertile, respectively
Bernstein 2020 [72]	Canada Apr/1995—Mar/2018	Biologic therapy					No difference for biologic therapy across socioeconomic status; p-values not reported

^a^all risk estimates reported for males compared with females. Risk estimates for socioeconomic variables are reported in key findings

Six studies (9%) examined adherence to biological treatment, and all reported statistically significantly lower adherence to biologics among female compared to male patients [45, 93–97].

None of the 4 studies (6%) examining income-related differences in the use of biological treatment [60, 72, 91, 92] reported a statistically significant association. One study [92] found an association between the highest level of education (master’s degree or higher) and a lower likelihood of biological treatment compared to the lowest education (college or less) in CD patients and overall IBD. One study (1.5%) examined educational differences in adherence to biological treatment and found no statistically significant association between educational status and adherence [97] (Table 4).

Meta-analysis was not relevant to studies reporting on corticosteroids and biological treatment due to the considerable variation in study designs and reporting of effect size.

### Meta-regression

In studies of surgery, meta-regression of the year of publication revealed a tendency towards smaller differences between males and females, the more recent the publication, although this trend was not statistically significant (Additional file 5: Figure A1). Measures of heterogeneity were lower in the meta-regression (*I*^*2*^ = 75.51 [54.75;95.18]% and ${{\varvec{\tau}}}^{2}$ = 0.08 [0.03;0.49]) than in the main meta-analysis (*I*^*2*^ = 86.19 [54.73;94.93]% and ${{\varvec{\tau}}}^{2}$ = 0.15 [0.03;0.45]), although not substantially different. Thus, this could indicate that the year of publication may explain some of the overall between-study heterogeneity in the main RE model of surgery.

In the studies of hospitalization, meta-regression of the year of publication revealed a tendency towards an increasing difference between males and females in the likelihood of hospitalization the more recent the publication, although not statistically significant (Additional file 5: Figure A2). The between-study heterogeneity was slightly lower (*I*^*2*^ = 84.57 [62.03;96.08]% and ${{\varvec{\tau}}}^{2}$ = 0.10 [0.03;0.44]) compared to the heterogeneity in the main meta-analysis (see above). These results do not indicate a significant effect of publication year on the between-study heterogeneity for hospitalization.

### Sensitivity analysis and publication bias

In sensitivity analyses comparing studies of univariate analyses with studies of multivariate analyses, we found that the overall heterogeneity was high in both RE models for surgery (Additional file 5: Figure A3). In contrast, the univariate model had a very low heterogeneity compared to the multivariate model of studies of hospitalization (Additional file 5: Figure A4). Thus, excluding univariate studies would not lower the heterogeneity, probably due to the high heterogeneity of the covariates included in the multivariate models, which made them less comparable, and/or due to unadjusted confounding.

Inspection of funnel plots indicated no significant risk of publication bias (Additional file 5: Figure A5 and Figure A6).

### Study quality assessment

All included cohort or case–control studies (*N* = 53) scored between 5 and 9 on the Newcastle–Ottawa Scale for quality assessment, with 88.7% of these studies scoring 7 or above, indicating a high-quality methodology (Additional file 3: Table A3). However, in total, 16 studies (23.9% of all included studies) reported only descriptive statistics (*N* (%), *p*-value) and no relative risk estimates, lowering the quality. Furthermore, the quality of evidence was lowered in the GRADE assessment to “very low” due to high inconsistency and indirectness when comparing outcomes across studies (Additional file 4: Table A4).

## Discussion

This systematic review identified 67 studies reporting the impact of sex (65 studies (97.0%)), income (10 studies (14.9%)) and education (6 studies (9%)) on the likelihood of bowel surgery, hospitalization, and use of corticosteroids and biologics in patients with IBD. In the meta-analysis, we observed the male sex to be significantly associated with a higher likelihood of bowel surgery, whereas sex did not impact the likelihood of hospitalization. Evidence of education- and income-related differences was minimal, with only a few studies identified for all four types of healthcare utilization, each pointing in a different direction. There was a high degree of heterogeneity among the studies, which we attempted to account for in the subgroup analyses and meta-regression.

### Sex and likelihood of surgery and hospitalization

In CD patients, sex had an influence on the likelihood of hospitalization, with a higher likelihood among men, but not on surgery, whereas in UC patients, sex was associated with surgery, with a higher likelihood among men, but it was not associated with hospitalization. Although sex-based differences were found, many studies reported no statistically significant sex differences in either the likelihood of surgery or hospitalization [26, 34, 36–38, 41–43, 45, 46, 48, 49, 51, 54, 57, 59–61, 65, 68, 71, 76–80, 84]. Similar results were observed in a review by Rustgi et al., that reported males generally undergo more IBD-related surgery than females [12], yet another study from the review by Rustgi et al. revealed higher probabilities among female patients [27].

### Sex and use of corticosteroids and biologics

In this review, only 2 of the 13 studies found statistically significant sex-based differences in corticosteroids, and with contradictive results [64, 89]. No studies examining sex and adherence to corticosteroids were identified. Contrary to this, all 6 studies of adherence to biologics and 2 of 11 studies examining the use of biologics found a statistically significantly lower adherence or lower probability of females using biologics compared to males [45, 64, 92–97]. These findings are similar to previous reviews [13, 15], which reported that the female sex was one of the most reliable predictors of non-adherence, and males were more likely to receive systemic treatments (including biologics) than females. A possible explanation may be a poorer response and effect of biologics in female patients, as reported in cohort studies [98, 99], that could affect adherence to medical treatment.

### Possible sex biases influencing the findings

Evidence is accumulating of underlying biological mechanisms explaining sex-based differences in IBD, including the association between sex hormones with susceptibility to IBD, the severity of symptoms, and disease progression [13, 100–104]. One study in this review suggested that more frequent ileal involvement in men with IBD has resulted in more bowel resections in men compared to women [26]. Moreover, there is evidence that males have a higher rate of colorectal cancer than females]13], which may also have contributed to differences in the likelihood of surgery between males and females. Finally, the observed higher likelihood of hospitalization may be associated with a higher likelihood of surgery. Shared decision-making is an important part of the therapeutic strategy for IBD and gives patients input into decisions about their care, including whether to have surgery or start medical treatment. Thus, patient preferences and concerns have a great impact on decision-making. For example, some women may be more likely to avoid a colectomy due to concerns about body image or future fertility after surgery [105]. Having surgery and receiving medical treatment should, thus, not solely be considered risks. It may also be a risk not to have surgery or delay medical treatment. If a lack of or delay of surgery or medical treatment negatively affects IBD worsening the clinical trajectory, then it could indicate undertreatment and health inequity. Finally, several studies in this review still report no statistically significant sex differences.

### Income and education and the likelihood of surgery and hospitalization

The impact of socioeconomic status often varies across studies due to different definitions of socioeconomic status. In this review, income and education were used as socioeconomic measures. However, the definition of income and education also varied across studies, resulting in very contradictive findings among the 9 included studies of associations between income and education and the likelihood of surgery or hospitalization [33, 38, 44, 60, 72–74, 81, 83]. Furthermore, the included studies were not directly comparable to findings from a recently published systematic review [14], which included measurements of socioeconomic status based on medical insurance.

### Income and education and use of corticosteroids and biologics

Very few studies examined income and education-related differences in using corticosteroids and biologics. One study reported that those with a lower income were more likely to take corticosteroids [72], and another study found an association between the highest education level and biological treatment use [92]. Finally, other studies reported no statistically significant differences. Sewell et al., also reported varying results, with one study from the review reporting no statistically significant differences in the use of steroids between deprived and non-deprived [18], and two studies reporting no significant income or educational differences in adherence to medical therapy generally [22, 106].

### Possible socioeconomic biases influencing the findings

Studies have linked low income and education to an increased likelihood of developing severe disease outcomes in various chronic disorders, suggesting underlying explanatory factors such as more severe chronic disease among individuals with low education are due to an unhealthier lifestyle [107–111]. Moreover, many of these were conducted in countries with healthcare access based on insurance status and non-universal healthcare coverage. Therefore, the reported differences concerning income and education may suggest that income and education are more of an indicator of lower access to surgical treatment than a reflection of a biologically lower likelihood of requiring surgery. However, there is currently no well-founded explanation for the observed differences.

### Strengths and limitations of the evidence included and of the review processes used

A major strength of this review is the unification of studies on sex, income, and education as socio-demographic predictors for IBD healthcare. A major strength is also the discussion of evidence from studies included in the meta-analysis and studies not eligible for inclusion. Furthermore, only studies with the same study design (retrospective cohort studies) and effect size measure were included in the meta-analysis to ensure comparability. In addition, a systematic approach was used based on the PRISMA guidelines for systematic reviews, and the choice of search terms was made with the guidance of an experienced health sciences librarian and lastly, screening and data extraction were performed independently by two persons.

A major limitation of this review is the lack of evidence for explaining observed differences. For sex, it may be a mixture of biological factors and individual health behavior. Another limitation is the low-GRADE score for the outcomes and the degree of between-study heterogeneity. The subgroup analyses revealed that the type of IBD, age group of patients, the country where the study was conducted, and publication year could explain some of this heterogeneity. The high heterogeneity is, in part, due to the broad inclusion/exclusion criteria used in the systematic literature search, such as the inclusion of both children and adults, all countries, and varying definitions of surgery, hospitalization, and use of medical treatment.

It is also a limitation that none of the included studies had clearly defined sex or gender. The use of sex/gender varied across the included studies, with 37 studies (55.2%) using the word “sex” while 11 studies (16.4%) used the word “gender”, and 19 studies (28.4%) using a mixture of both words. There were only three studies that defined sex as “biological sex” and gender as a socially constructed definition. Thus, for this review, sex and gender were combined and one-word “sex” was used to describe biological sex. Lastly, we found a minimal number of studies examining the association between educational and income-related differences and all four types of healthcare utilization. This lack of studies could be due to the search terms. For example, “education” was considered too broad and not included in the search block. In addition, we excluded studies using surrogate measures for socioeconomic status, such as insurance status, as, in many countries, healthcare is not based on a healthcare insurance system.

## Conclusion

This systematic review provides a comprehensive update of our knowledge of sex-, income-, and education-based differences in IBD. The review reports on results from high-quality cohort studies showing that male patients are more likely to have surgery and be hospitalized than female patients with IBD, although only significant for surgery in the meta-analysis. In contrast, the evidence of sex-, income-, and education-based differences for using corticosteroids or biologics was sparse, except for adherence to biologics, where high-quality studies found statistically significantly lower adherence to biologics among female patients compared to male patients. Nevertheless, the review revealed considerable heterogeneity between the studies. In summary, this review underlines that it is important to consider IBD patients’ sociodemographic characteristics, especially sex, in both future studies and clinical practice, since they can be significant explanatory variables and/or confounders for healthcare outcomes and patients may have different needs for social support in relation to the shared healthcare decision-making process. Although it could be argued that sex per se may influence the severity of IBD and hence the assessed outcomes, there is no obvious explanation for the income- and education-based differences. However, the impact of patient preferences, health literacy, healthcare systems, and differential access to care by sex and socioeconomic status on patient outcomes should not be underestimated. Identifying such underlying associations will provide policymakers with an evidence base to target interventions against inequality in the use of healthcare.

### Supplementary Information

Additional file 1: Table A1. Search terms. 

Additional file 2: Table A2. Background characteristics of included studies.

Additional file 3: Table A3. Newcastle-Ottawa quality assessment.

Additional file 4: Table A4. GRADE evidence profile of risk of surgery and hospitalization.

Additional file 5: Figure A1. Meta-regression of log HRs for risk of surgery by study publication year. Figure A2. Meta-regression of log ORs for risk of hospitalization by study publication year. Figure A3. Forest plot of subgroup (univariate/multivariate) meta-analysis of HRs for risk of surgery. Figure A4. Forest plot of subgroup (univariate/multivariate) meta-analysis of ORs for risk of hospitalization. Figure A5. Funnel plot of the meta-analysis of published studies on surgery. Figure A6. Funnel plot of the meta-analysis of published studies on hospitalization.

## Data Availability

All data generated or analyzed during this study are included in this published article (and its supplementary information files).

## Abbreviations

IBD: Inflammatory bowel disease
CD: Crohn’s disease
UC: Ulcerative colitis
5-ASA: 5-Aminosalicylic acid
PRISMA: Preferred Reporting Items for Systematic reviews and Meta-Analyses
RR: Relative risk
OR: Odd ratio
HR: Hazard ratio
NOS: Newcastle-Ottawa Scale
GRADE: Grading of Recommendations, Assessment, Development and Evaluations
RE: Random effects
EE: Equal effects
CI: Confidence interval
