# Supplementary material for: Impact of sex and socioeconomic status on the likelihood of surgery, hospitalization, and use of medications in inflammatory bowel disease: a systematic review and meta-analysis
Source: Syst Rev. 2024 Jun 24;13:164. doi: 10.1186/s13643-024-02584-3 (PMC11194997; doi:10.1186/s13643-024-02584-3)
Supplement: Supplementary file 1 — Additional file 1: Table A1. Search terms. [file 13643_2024_2584_MOESM1_ESM.docx]

**Table A1. Search terms**

**EMBASE**

- ***Gender and IBD***

| 1. | exp gender equity/ |
| --- | --- |
| 2. | exp gender inequality/ |
| 3. | sex difference/ |
| 4. | "Gender equalit*".ti,ab,kw. |
| 5. | "Gender inequalit*".ti,ab,kw. |
| 6. | "Gender disparit*".ti,ab,kw. |
| 7. | "Gender inequit*".ti,ab,kw. |
| 8. | "Gender difference".ti,ab,kw. |
| 9. | "Sex difference".ti,ab,kw. |
| 10. | "Sex disparit*".ti,ab,kw. |
| 11. | "Sex inequalit*".ti,ab,kw. |
| 12. | "Sex inequit*".ti,ab,kw. |
| 13. | ("gender‐based" or "gender‐related" or "gender differences" or "gender factors").ti,ab,kw. |
| 14. | ((sex or gender) adj3 (analysis or specific or difference? or factor? or inequit* or disparit* or inequalit*)).ti,ab,kw. |
| 15. | exp sex factor/ |
| 16. | 1 or 2 or 3 or 4 or 5 or 6 or 7 or 8 or 9 or 10 or 11 or 12 or 13 or 14 or 15 |
| 17. | Colitis*.ti,ab,kw. |
| 18. | Crohn*.ti,ab,kw. |
| 19. | (ulcer* adj1 colitis).ti,ab,kw. |
| 20. | Ulcerative colitis*.ti,ab,kw. |
| 21. | IBD.ti,ab,kw. |
| 22. | inflammatory bowel disease.ti,ab,kw. |
| 23. | exp inflammatory bowel disease/ |
| 24. | 17 or 18 or 19 or 20 or 21 or 22 or 23 |
| 25. | 16 and 24 |
| 26. | limit 25 to yr="2012 - 2024" |
|  | **RESULT: 2,742** |

- ***Socioeconomic status and IBD***

| 1. | exp inflammatory bowel disease/ |
| --- | --- |
| 2. | Colitis*.ti,ab,kw. |
| 3. | Crohn*.ti,ab,kw. |
| 4. | (ulcer* adj1 colitis).ti,ab,kw. |
| 5. | Ulcerative colitis*.ti,ab,kw. |
| 6. | IBD.ti,ab,kw. |
| 7. | inflammatory bowel disease.ti,ab,kw. |
| 8. | 1 or 2 or 3 or 4 or 5 or 6 or 7 |
| 9. | Academic achievement/ |
| 10. | economic status/ |
| 11. | exp Educational status/ |
| 12. | exp employment/ |
| 13. | exp employment status/ |
| 14. | exp unemployment/ |
| 15. | exp family income/ |
| 16. | exp highest income group/ |
| 17. | exp household income/ |
| 18. | exp Income group/ |
| 19. | exp Income inequality/ |
| 20. | Income/ |
| 21. | exp lowest income group/ |
| 22. | exp middle income group/ |
| 23. | exp personal income/ |
| 24. | exp Social determinants of health/ |
| 25. | Health equity/ |
| 26. | Health disparity/ |
| 27. | Health care disparity/ |
| 28. | Social class/ |
| 29. | Social status/ |
| 30. | socioeconomics/ |
| 31. | "Demographic characteristic*".ti,ab,kw. |
| 32. | "Demographic status".ti,ab,kw. |
| 33. | Deprived.ti,ab,kw. |
| 34. | Disadvantaged.ti,ab,kw. |
| 35. | "Economic Factor*".ti,ab,kw. |
| 36. | "Economic inequality".ti,ab,kw. |
| 37. | "Economic status".ti,ab,kw. |
| 38. | "Educational level*".ti,ab,kw. |
| 39. | "Educational status".ti,ab,kw. |
| 40. | "Employment status".ti,ab,kw. |
| 41. | "Highest income group".ti,ab,kw. |
| 42. | Income*.ti,ab,kw. |
| 43. | Income disparit*.ti,ab,kw. |
| 44. | "Income group*".ti,ab,kw. |
| 45. | "Income inequalit*".ti,ab,kw. |
| 46. | "Income inequit*".ti,ab,kw. |
| 47. | "Income level*".ti,ab,kw. |
| 48. | "Lowest income group".ti,ab,kw. |
| 49. | Poverty.ti,ab,kw. |
| 50. | Occupation*.ti,ab,kw. |
| 51. | "Salar*".ti,ab,kw. |
| 52. | "Social background*".ti,ab,kw. |
| 53. | "Social Class*".ti,ab,kw. |
| 54. | "Social economic status".ti,ab,kw. |
| 55. | "Social Factor*".ti,ab,kw. |
| 56. | "Social rank*".ti,ab,kw. |
| 57. | "Social standing*".ti,ab,kw. |
| 58. | "Social state*".ti,ab,kw. |
| 59. | "Social status*".ti,ab,kw. |
| 60. | Sociodemographic*.ti,ab,kw. |
| 61. | "Sociodemographic factor*".ti,ab,kw. |
| 62. | "Socioeconomic difference*".ti,ab,kw. |
| 63. | "Social determinant*".ti,ab,kw. |
| 64. | "Socioeconomic factor*".ti,ab,kw. |
| 65. | "Socioeconomic group*".ti,ab,kw. |
| 66. | Socioeconomic status*.ti,ab,kw. |
| 67. | Socio-economic status*.ti,ab,kw. |
| 68. | "Socioeconomic*".ti,ab,kw. |
| 69. | "Health inequit*".ti,ab,kw. |
| 70. | "Health status disparit*".ti,ab,kw. |
| 71. | "Healthcare disparit*".ti,ab,kw. |
| 72. | "family income".ti,ab,kw. |
| 73. | "highest income group".ti,ab,kw. |
| 74. | "household income".ti,ab,kw. |
| 75. | "Lowest income group".ti,ab,kw. |
| 76. | "personal income".ti,ab,kw. |
| 77. | (Socioeconomic adj2 inequalit*).ti,ab,kw. |
| 78. | (Socioeconomic adj2 inequit*).ti,ab,kw. |
| 79. | (Socioeconomic adj2 disparit*).ti,ab,kw. |
| 80. | ((poverty or "low‐income" or socioeconomic$ or social) adj2 (analysis or disadvantage$ or specific or difference? or factor? or inequalit$ or depriv$ or inequit$ or disparit$)).ti,ab,kw. |
| 81. | 9 or 10 or 11 or 12 or 13 or 14 or 15 or 16 or 17 or 18 or 19 or 20 or 21 or 22 or 23 or 24 or 25 or 26 or 27 or 28 or 29 or 30 or 31 or 32 or 33 or 34 or 35 or 36 or 37 or 38 or 39 or 40 or 41 or 42 or 43 or 44 or 45 or 46 or 47 or 48 or 49 or 50 or 51 or 52 or 53 or 54 or 55 or 56 or 57 or 58 or 59 or 60 or 61 or 62 or 63 or 64 or 65 or 66 or 67 or 68 or 69 or 70 or 71 or 72 or 73 or 74 or 75 or 76 or 77 or 78 or 79 or 80 |
| 82. | 8 and 81 |
| 83. | limit 82 to yr="2012 - 2024" |
|  | **RESULT: 4,349** |

**MEDLINE**

- ***Gender and IBD***

| 1. | exp gender equity/ |
| --- | --- |
| 2. | "Gender equalit*".ti,ab,kw. |
| 3. | "Gender inequalit*".ti,ab,kw. |
| 4. | "Gender disparit*".ti,ab,kw. |
| 5. | "Gender inequit*".ti,ab,kw. |
| 6. | "Gender difference".ti,ab,kw. |
| 7. | "Sex difference".ti,ab,kw. |
| 8. | "Sex disparit*".ti,ab,kw. |
| 9. | "Sex inequalit*".ti,ab,kw. |
| 10. | "Sex inequit*".ti,ab,kw. |
| 11. | ((gender or sex) adj3 (analysis or specific or difference? or factor? or inequit$ or disparit$ or inequalit$)).ti,ab,kw. |
| 12. | ("gender‐based" or "gender‐related" or "gender differences" or "gender factors").ti,ab,kw. |
| 13. | exp Sex Factors/ |
| 14. | 1 or 2 or 3 or 4 or 5 or 6 or 7 or 8 or 9 or 10 or 11 or 12 or 13 |
| 15. | Colitis*.ti,ab,kw. |
| 16. | Crohn*.ti,ab,kw. |
| 17. | (ulcer* adj1 colitis).ti,ab,kw. |
| 18. | Ulcerative colitis*.ti,ab,kw. |
| 19. | IBD.ti,ab,kw. |
| 20. | inflammatory bowel disease.ti,ab,kw. |
| 21. | exp inflammatory bowel disease/ |
| 22. | 15 or 16 or 17 or 18 or 19 or 20 or 21 |
| 23. | 14 and 22 |
| 24. | limit 23 to yr="2012 - 2024" |
|  | **RESULT: 1,191** |

- ***Socioeconomic status and IBD***

| 1. | economic status/ |
| --- | --- |
| 2. | exp Educational status/ |
| 3. | exp employment/ |
| 4. | exp unemployment/ |
| 5. | Income/ |
| 6. | exp Social determinants of health/ |
| 7. | exp sociodemographic factors/ |
| 8. | Health equity/ |
| 9. | Health inequities/ |
| 10. | Healthcare disparities/ |
| 11. | Health status disparities/ |
| 12. | Social class/ |
| 13. | Social status/ |
| 14. | exp socioeconomic factors/ |
| 15. | "Demographic characteristic*".ti,ab,kw. |
| 16. | "Demographic status".ti,ab,kw. |
| 17. | Deprived.ti,ab,kw. |
| 18. | Disadvantaged.ti,ab,kw. |
| 19. | "Economic Factor*".ti,ab,kw. |
| 20. | "Economic inequality".ti,ab,kw. |
| 21. | "Economic status".ti,ab,kw. |
| 22. | "Educational status".ti,ab,kw. |
| 23. | "Education* level".ti,ab,kw. |
| 24. | "Employment status".ti,ab,kw. |
| 25. | "Highest income group".ti,ab,kw. |
| 26. | Income*.ti,ab,kw. |
| 27. | Income disparit*.ti,ab,kw. |
| 28. | "Income group*".ti,ab,kw. |
| 29. | "Income inequalit*".ti,ab,kw. |
| 30. | "Income inequit*".ti,ab,kw. |
| 31. | "Income level*".ti,ab,kw. |
| 32. | "Lowest income group".ti,ab,kw. |
| 33. | Poverty.ti,ab,kw. |
| 34. | Occupation*.ti,ab,kw. |
| 35. | "Salar*".ti,ab,kw. |
| 36. | "Social background*".ti,ab,kw. |
| 37. | "Social Class*".ti,ab,kw. |
| 38. | "Social economic status".ti,ab,kw. |
| 39. | "Social Factor*".ti,ab,kw. |
| 40. | "Social rank*".ti,ab,kw. |
| 41. | "Social standing*".ti,ab,kw. |
| 42. | "Social state*".ti,ab,kw. |
| 43. | "Social status*".ti,ab,kw. |
| 44. | Sociodemographic*.ti,ab,kw. |
| 45. | "Sociodemographic factor*".ti,ab,kw. |
| 46. | "Socioeconomic difference*".ti,ab,kw. |
| 47. | "Social determinant*".ti,ab,kw. |
| 48. | "Socioeconomic factor*".ti,ab,kw. |
| 49. | "Socioeconomic group*".ti,ab,kw. |
| 50. | Socioeconomic status*.ti,ab,kw. |
| 51. | Socio-economic status*.ti,ab,kw. |
| 52. | "Socioeconomic*".ti,ab,kw. |
| 53. | "Health inequit*".ti,ab,kw. |
| 54. | "Health status disparit*".ti,ab,kw. |
| 55. | "Healthcare disparit*".ti,ab,kw. |
| 56. | "family income".ti,ab,kw. |
| 57. | "highest income group".ti,ab,kw. |
| 58. | "household income".ti,ab,kw. |
| 59. | "Lowest income group".ti,ab,kw. |
| 60. | "personal income".ti,ab,kw. |
| 61. | (Socioeconomic adj2 inequalit*).ti,ab,kw. |
| 62. | (Socioeconomic adj2 inequit*).ti,ab,kw. |
| 63. | (Socioeconomic adj2 disparit*).ti,ab,kw. |
| 64. | ((poverty or "low‐income" or socioeconomic$ or social) adj2 (analysis or disadvantage$ or specific or difference? or factor? or inequalit$ or depriv$ or inequit$ or disparit$)).ti,ab,kw. |
| 65. | 1 or 2 or 3 or 4 or 5 or 6 or 7 or 8 or 9 or 10 or 11 or 12 or 13 or 14 or 15 or 16 or 17 or 18 or 19 or 20 or 21 or 22 or 23 or 24 or 25 or 26 or 27 or 28 or 29 or 30 or 31 or 32 or 33 or 34 or 35 or 36 or 37 or 38 or 39 or 40 or 41 or 42 or 43 or 44 or 45 or 46 or 47 or 48 or 49 or 50 or 51 or 52 or 53 or 54 or 55 or 56 or 57 or 58 or 59 or 60 or 61 or 62 or 63 or 64 |
| 66. | Colitis*.ti,ab,kw. |
| 67. | Crohn*.ti,ab,kw. |
| 68. | (ulcer* adj1 colitis).ti,ab,kw. |
| 69. | Ulcerative colitis*.ti,ab,kw. |
| 70. | IBD.ti,ab,kw. |
| 71. | inflammatory bowel disease.ti,ab,kw. |
| 72. | exp inflammatory bowel disease/ |
| 73. | 66 or 67 or 68 or 69 or 70 or 71 or 72 |
| 74. | 65 and 73 |
| 75. | limit 74 to yr="2012 - 2024" |
|  | **RESULT: 1,182** |

**CINAHL**

- ***Gender and IBD***

| 1. | (MH "Inflammatory Bowel Diseases+") |
| --- | --- |
| 2. | IBD |
| 3. | Crohn* |
| 4. | Ulcerative colitis |
| 5. | Inflammatory bowel disease |
| 6. | 1 or 2 or 3 or 4 or 5 |
| 7. | MH Gender equality |
| 8. | Gender |
| 9. | Gender equality |
| 10. | Gender inequality |
| 11. | Gender disparity |
| 12. | Gender inequity |
| 13. | Gender difference |
| 14. | Sex difference |
| 15. | Sex disparity |
| 16. | Sex inequality |
| 17. | Sex inequity |
| 18. | 7 or 8 or 9 or 10 or 11 or 12 or 13 or 14 or 15 or 16 or 17 |
| 19. | 6 and 18 |
|  | Limiters - Published Date: 20120101-20241231 |
|  | **RESULT: 456** |

- ***Socioeconomic status and IBD***

| 1. | (MH "Inflammatory Bowel Diseases+") |
| --- | --- |
| 2. | IBD |
| 3. | Crohn* |
| 4. | Ulcerative colitis |
| 5. | Inflammatory bowel disease |
| 6. | 1 or 2 or 3 or 4 or 5 |
| 7. | (MH "Occupations and Professions") |
| 8. | (MH "Income") |
| 9. | (MH "Social Determinants of Health") |
| 10. | (MH "Socioeconomic Factors+") |
| 11. | disadvantaged |
| 12. | deprived |
| 13. | demographic status |
| 14. | demographic characteristics |
| 15. | employment status |
| 16. | employment |
| 17. | educational status |
| 18. | educational level |
| 19. | economic status |
| 20. | economic inequalit* |
| 21. | economic factor* |
| 22. | income |
| 23. | occupation |
| 24. | poverty |
| 25. | salary |
| 26. | socioeconomic* |
| 27. | sociodemographic* |
| 28. | social rank* |
| 29. | social state* |
| 30. | social standing |
| 31. | social inequalit* |
| 32. | social factors |
| 33. | social economic status |
| 34. | social class |
| 35. | social background |
| 36. | personal income |
| 37. | middle income group |
| 38. | lowest income group |
| 39. | household income |
| 40. | highest income group |
| 41. | family income |
| 42. | healthcare disparit* |
| 43. | health status disparit* |
| 44. | health inequit* |
| 45. | 7 or 8 or 9 or 10 or 11 or 12 or 13 or 14 or 15 or 16 or 17 or 18 pr 19 or 20 or 21 or 22 or 23 or 24 or 25 or 26 or 27 or 28 or 29 or 30 or 31 or 32 or 33 or 34 or 35 or 36 or 37 or 38 or 39 or 40 or 41 or 42 or 43 or 44 |
| 46. | 6 and 45 |
|  | Limiters - Published Date: 20120101-20241231 |
|  | **RESULT: 551** |
|  |  |
|  |  |

**Web of Science**

- ***Gender and IBD***

| 1. | TI=(inflammatory bowel disease) | |
| --- | --- | --- |
| 2. | AB=(inflammatory bowel disease) | |
| 3. | TI=(IBD) | |
| 4. | AB=(IBD) | |
| 5. | TI=(Crohn*) | |
| 6. | AB=(Crohn*) | |
| 7. | TI=(CD) | |
| 8. | AB=(CD) | |
| 9. | TI=(ulcerative colitis) | |
| 10. | AB=(ulcerative colitis) | |
| 11. | TI=(colitis*) | |
| 12. | AB=(colitis*) | |
| 13. | TI=(UC) | |
| 14. | AB=(UC) | |
| 15. | 1 or 2 or 3 or 4 or 5 or 6 or 7 or 8 or 9 or 10 or 11 or 12 or 13 or 14 | |
| 16. | TI Gender.ti,ab or AB gender.ti,ab | |
| 17. | TI "Gender equality" or AB "Gender equality" | |
| 18. | TI "Gender inequality" or AB "gender inequality" | |
| 19. | TI "Gender disparity" or AB "gender disparity" | |
| 20. | TI "Gender inequity" or AB "gender inequity" | |
| 21. | TI "Gender difference" or AB "Gender difference" | |
| 22. | TI "Sex difference" or AB "Sex difference" | |
| 23. | TI "Sex disparity" or AB "Sex disparity" | |
| 24. | TI "Sex inequality" or AB "Sex inequality" | |
| 25. | TI "Sex inequity" or AB "Sex inequity" | |
| 26. | 16 or 17 or 18 or 19 or 20 or 21 or 22 or 23 or 24 or 25 | |
| 27. | 15 and 26 and 2020 or 2021 or 2019 or 2018 or 2017 or 2016 or 2015 or 2014 or 2013 or 2012 (Publication Years) | |
| **RESULT: 2,186** | |  |
|  | |  |

- ***Socioeconomic status and IBD***

| 1. | TI=(inflammatory bowel disease) |
| --- | --- |
| 2. | AB=(inflammatory bowel disease) |
| 3. | TI=(IBD) |
| 4. | AB=(IBD) |
| 5. | TI=(Crohn*) |
| 6. | AB=(Crohn*) |
| 7. | TI=(CD) |
| 8. | AB=(CD) |
| 9. | TI=(ulcerative colitis) |
| 10. | AB=(ulcerative colitis) |
| 11. | TI=(colitis*) |
| 12. | AB=(colitis*) |
| 13. | TI=(UC) |
| 14. | AB=(UC) |
| 15. | 1 or 2 or 3 or 4 or 5 or 6 or 7 or 8 or 9 or 10 or 11 or 12 or 13 or 14 |
| 16. | TI="demographic characteristics" or AB="demographic characteristics" |
| 17. | TI="demographic status" or AB="demographic status" |
| 18. | TI=Demography or AB=demography |
| 19. | TI=Deprived or AB=deprived |
| 20. | TI=Disadvantaged or AB=disadvantaged |
| 21. | TI="Economic Factors" or AB="Economic Factors" |
| 22. | TI="Economic inequality" or AB="Economic inequality" |
| 23. | TI="Economic Status" or AB="Economic Status" |
| 24. | TI=Education or AB=Education |
| 25. | TI="Educational level" or AB="Educational level" |
| 26. | TI="Educational status" or AB="Educational status" |
| 27. | TI=Employment or AB=Employment |
| 28. | TI="Employment status" or AB="Employment status" |
| 29. | TI="Highest income group" or AB="Highest income group" |
| 30. | TI=Income or AB=Income |
| 31. | TI="Income disparity" or AB="Income disparity" |
| 32. | TI="Income group" or AB="Income group" |
| 33. | TI="Income inequality" or AB="Income inequality" |
| 34. | TI="Income inequity" or AB="Income inequity" |
| 35. | TI="Income level" or AB="Income level" |
| 36. | TI="Lowest income group" or AB="Lowest income group" |
| 37. | TI=Occupation* or AB=occupation* |
| 38. | TI=profession* or AB=profession* |
| 39. | TI=Poverty or AB=Poverty |
| 40. | TI=Salary or AB=Salary |
| 41. | TI="Social background" or AB="Social background" |
| 42. | TI="Social Class" or AB="Social Class" |
| 43. | TI="Social economic status" or AB="Social economic status" |
| 44. | TI="Social Factors" or AB="Social Factors" |
| 45. | TI="Social inequality" or AB="Social inequality" |
| 46. | TI="Social rank" or AB="Social rank" |
| 47. | TI="Social standing" or AB="Social standing" |
| 48. | TI="Social state" or AB="Social state" |
| 49. | TI="Social status" or AB="Social status" |
| 50. | TI=Sociodemographic or AB=Sociodemographic |
| 51. | TI="Sociodemographic factors" or AB="Sociodemographic factors" |
| 52. | TI="Socioeconomic difference" or AB="Socioeconomic difference" |
| 53. | TI="Social determinant" or AB="Social determinant" |
| 54. | TI="Socioeconomic disparity" or AB="Socioeconomic disparity" |
| 55. | TI="Socioeconomic factors" or AB="Socioeconomic factors" |
| 56. | TI="Socioeconomic group" or AB="Socioeconomic group" |
| 57. | TI="Socioeconomic inequality" or AB="Socioeconomic inequality" |
| 58. | TI="Socioeconomic inequity" or AB="Socioeconomic inequity" |
| 59. | TI="Socioeconomic status" or AB="Socioeconomic status" |
| 60. | TI="Socio-economic status" or AB="Socio-economic status" |
| 61. | TI=Socioeconomics or AB=Socioeconomics |
| 62. | TI="Health inequit*" or AB="Health inequit*" |
| 63. | TI="Health status disparit*" or AB="Health status disparit*" |
| 64. | TI="Healthcare disparit*" or AB="Healthcare disparit*" |
| 65. | TI="family income" or AB= "family income" |
| 66. | TI="highest income group" or AB="highest income group" |
| 67. | TI="household income" or AB="household income" |
| 68. | TI="Lowest income group" or AB="Lowest income group" |
| 69. | TI="middle income group" or AB="middle income group" |
| 70. | TI="personal income" or AB="personal income" |
| 71. | 16 or 17 or 18 or 19 or 20 or 21 or 22 or 23 or 24 or 25 or 26 or 27 or 28 or 29 or 30 or 31 or 32 or 33 or 34 or 35 or 36 or 37 or 38 or 39 or 40 or 41 or 42 or 43 or 44 or 45 or 46 or 47 or 48 or 49 or 50 or 51 or 52 or 53 or 54 or 55 or 56 or 57 or 58 or 59 or 60 or 61 or 62 or 63 or 64 or 65 or 66 or 67 or 68 or 69 or 70 |
| 72. | 15 and 71 and 2020 or 2021 or 2019 or 2018 or 2017 or 2016 or 2015 or 2014 or 2013 or 2012 (Publication Years) |
|  | **RESULT: 2,237** |
