# Supplementary material for: Impact of sex and socioeconomic status on the likelihood of surgery, hospitalization, and use of medications in inflammatory bowel disease: a systematic review and meta-analysis
Source: Syst Rev. 2024 Jun 24;13:164. doi: 10.1186/s13643-024-02584-3 (PMC11194997; doi:10.1186/s13643-024-02584-3)
Supplement: Supplementary file 2 — Additional file 2: Table A2. Background characteristics of included studies. [file 13643_2024_2584_MOESM2_ESM.docx]

**Table A2. Background characteristics of included studies**

| **Author and year** | **Country and study period** | **Study design and setting** | **Data source** | **Number of patients, N (%)** | | | | **Sex** | **SES** | **Definition of SES** | **Children/**  **adults** | **Outcome studied** |
| --- | --- | --- | --- | --- | --- | --- | --- | --- | --- | --- | --- | --- |
|  |  |  |  | **IBD** | **CD** | **UC** | **Males** |  |  |  |  |  |
| Calvo-Arbeloa 2020 | Spain  Jan/2019 - Jun/2019 | Cross-sectional observational study, tertiary care hospital | Dispensation records of the pharmacy department of a tertiary care hospital. | 178 | - | - | 107 (60.1) | Yes | Yes | No education; Primary; Secondary; University | Adults | Biologics |
| Khalili 2020 | Sweden  Jan/2014 - Dec/2014 | Retrospective cohort and cross-sectional study | National Patient Register | 29879 | 10117 (33.9) | 19762 (66.1) | 15358 (51.4) | Yes | Yes | level of education:  =< 9y, 10-12y, >12y | Adults | Biologics |
| Lagana 2019 | Italy,  Before 2019 | Retrospective multicenter study | Clinical records | 349 | - | - | 194 (55.6) | Yes | No |  | Adults | Biologics |
| Lin 2013 | USA  1998 - 2010 | Retrospective study | National survey data | 1137 | 769 (68) | 368 (32) | 498 (44) | Yes | No |  | Adults | Biologics |
| Mahlich 2018 | Japan  Survey data collected in Feb/2016 | Secondary data analysis of a nationwide web-based cross-sectional survey study | Nationwide web-based survey data | 1035 | 235 (23) | 800 (77) | 675 (65.2) | Yes | Yes | Highest education and household income in million Japanese Yen | Adults | Biologics |
| Rundquist 2018 | Sweden  2005 - 2017 | Retrospective cohort | The National Quality Registry for IBD in Sweden | 94 | 94 (100) | 0 (0) | 40 (42.6) | Yes | No |  | Children and adults | Biologics |
| Schultheiss 2019 | The Netherlands  Jan/2011 - Dec/2017 | Retrospective cohort study | The pharmacy's record of the University Medical Centre Utrecht | 529 | 408 (77.1) | 121 (22.9) | 264 (49.9) | Yes | No |  | Children and adults | Biologics |
| Tanaka 2018 | Japan  Between Oct/2010 and Dec/2013 | Retrospective, multicenter cohort study | Medical records | 1189 | 1189 (100) | 0 (0) | 826 (69.5) | Yes | No |  | Adults | Biologics |
| Barkan 2024 | Israel  - | Cross-sectional cohort study | Patient self-reported and nurse-reported questionnaires. | 337 | 262 (66) | 75 (34) | 217 (54) | Yes | No |  | Adults | Corticosteroids |
| daSilva 2015 | Brazil  Jan/2011 - Sep/2012 | Cross-sectional, IBD referral centers | Interviews and medical records | 267 | 0 (0) | 267 (100) | 87 (32.6) | Yes | No |  | Children and adults | Corticosteroids |
| Sundel 2024 | USA  Mar/2017 – Mar/2021 | Cross-sectional | National surgical quality database | 3143 | 1842 (59.5) | 1241 (40.1) | 1593 (51) | Yes | No |  | Adults | Corticosteroids |
| Lee 2012 | USA  May/2007 - May/2010 | Cross-sectional, multicenter patient registry | The ImproveCareNow Network patient registry | 1409 | 993 (70) | 416 (30) | 757 (54) | Yes | No |  | Only children (<18) | Corticosteroids; Biologics |
| Barnes 2017 | USA  2013 | Retrospective cohort | A nationwide readmissions database | 2733 | 1733 (63) | 1000 (37) | 1476 (54.0) | Yes | No |  | Only children (<18) | Hospitalization/inpatient visit |
| Chudy-Onwugaje 2021 | USA  Nov/2012 - Dec/2015 | Retrospective cohort, tertiary referral centers (outpatient IBD practices at University Medical School and University Medical Center) | Electronic medical records | 735 | 454 (61.8) | 281 (38.2) | 321 (44) | Yes | No |  | Children and adults | Hospitalization/inpatient visit |
| Gunnells 2015 | USA  2012 - 2013 | Retrospective cohort study | National Surgical Quality Improvement Program database | 2523 | 1855 (74) | 668 (26) | 1202 (48) | Yes | No |  | Adults | Hospitalization/inpatient visit |
| Limsrivilai 2017 | USA  Jul/2012 - Jun/2015 | A retrospective study of medical records, university hospital | Electronic medical records | 1.005 | 611 (60.8) | 394 (39.2) | 474 (47.2) | Yes | No |  | Adults | Hospitalization/inpatient visit |
| Mandel 2014 | Hungary  Jan/2008 - ? | Retrospective cohort | Medical records | 194 | 152 (78.4) | 42 (21.6) | 88 (45.4) | Yes | No |  | Adults | Hospitalization/inpatient visit |
| Micic 2017 | USA  ? - 2013 | Retrospective cohort | The Healthcare Cost and Utilization Project (HCUP) Nationwide Readmissions Database (NRD) 2013 | 43680 | 26770 (61.2) | 16908 (38.8) | 19361 (44.3) | Yes | Yes | Median income quartiles for patient's ZIP code (Q1-Q4) | Adults | Hospitalization/inpatient visit |
| Mudireddy 2017 | USA  Jan/2007 - Dec/2010 | Retrospective cohort study | Electronic medical record of a tertiary academic medical center | 439 | 295 (67) | 144 (33) | 198 (45) | Yes | No |  | Adults | Hospitalization/inpatient visit |
| Poojary 2017 | USA  2013 | Retrospective cohort, | National Readmissions Database | 26904 | 0 (0) | 26904 (100) | 11536 / 1472 (47.78 / 53.37) (No readmissions / At least one readmission) | Yes | Yes | Median household income category for patients ZIP code in percentiles | Adults | Hospitalization/inpatient visit |
| Reja 2020 | USA  Jan/2016 - Dec/2016 | multicenter retrospective cohort | The Nationwide Readmission Database (NRD) | 6506 | 4158 (63.9) | 2348 (36.1) | 2941 (45.2) | Yes | No |  | Adults | Hospitalization/inpatient visit |
| Axelrad 2019 | USA  Jan/2007 - Jun/2017 | Retrospective cohort, tertiary care center, patients in Colorectal Cancer Surveillance Colonoscopy Program | Electronic medical record | 947 | - | - | 484 (51.1) | Yes | No |  | Adults | Hospitalization/inpatient visit; Corticosteroids |
| AbouKhalil 2018 | Canada  Jan/1998-Dec/2004 and  Jan/2005-Dec/2011 | Retrospective pre/post cohort, provincial database study | Provincial health insurance agency - Regie d’Assurance Maladie du Quebec (RAMQ) | 6142 | 0 (0) | 6142 (100) | 3080 (50.1) | Yes | Yes | Economic deprivation (based on post-secondary education, employment, and average income): Low ; Moderate ; High | Adults | Surgery |
| Akintimehin 2018 | Ireland  Jan/2009 - Dec/2025 | Retrospective cohort, tertiary referral centre for paediatric gastroenterology | Hospital discharge coding system and hospital department patient database. | 55 | 0 (0) | 55 (100) | 23 (42) | Yes | No |  | Only children (<18) | Surgery |
| Chhay 2015 | UK  Jan/1989 - Dec/2009 | Population-based cohort study | The Clinical Practice Research Datalink CPRD - A primary care database containing anonymised patient records for approximately 8% of the UK population | 8673 | 0 (0) | 8673 (100) | 4507 (52.0) | Yes | No |  | Children and adults | Surgery |
| DeCristofaro 2022 | Italy  2010 - 2020 | Retrospective cohort | Medical records | 116 |  | 116 (100) | 59 (51) | Yes | No |  | Children and adults | Surgery |
| Eder 2017 | Poland  Jan/2014 - Dec/2015 | Secondary analysis of a prospective, multicentre, observational cohort study | Electronic database | 256 | 256 (100) | 0 (0) | 143 (55.9) | Yes | No |  | Adults | Surgery |
| Gao 2012 | China  Jan/2003 - Dec/2010 | Prospective cohort study | Local medical files | 323 | 323 (100) | 0 (0) | 213 (65.9) | Yes | No |  | Children and adults | Surgery |
| Goel 2013 | India  Jan/1995 - Dec/2008 | Retrospective cohort | Medical charts | 223 | 223 (100) | 0 (0) | 129 (57.9) | Yes | No |  | Adults | Surgery |
| Kim 2017 | South Korea  Mar/1987 - Dec/2013 | Retrospective cohort | National IBD registry; medical records; interviews | 594 | 594 (100) | 0 (0) | 421 (70.9) | Yes | No |  | Only children (<18) | Surgery |
| King 2020 | UK  Apr/2017 - Mar/2017 | Retrospective cohort | National administrative database | 10051 | 0 (0) | 10051 (100) | 5382 (54) | Yes | Yes | Deprivation based on income, employment, crime, and living environment, in quintiles, with 1 being the most deprived and 5 being the least deprived. | Adults | Surgery |
| Lee 2023 | USA  Oct/2015 – Dec/2019 | Retrospective cohort | National health database | 9555 | 6392 (66.9) | 3163 (33.1) | 4829 (50.5) | Yes | No |  | Adults | Surgery |
| Li 2015 | China  Sep/2010 - Aug/2014 | Retrospective study | Local hospital's electronic database and follow-up telephone calls. | 343 | 343 (100) | 0 (0) | 240 (70) | No | Yes | Basic education including no education at all and elementary school / Higher education including high school, higher vocational education, university, and higher degrees | Adults | Surgery |
| Magro 2019 | Portugal  2015 - 2016 | Prognostic model study, multicentre | Portuguese IBD group database | 1210 | 0 (0) | 1210 (100) | 540 (45) | Yes | No |  | Adults | Surgery |
| McLoughlin 2020 | USA  2006-2012 | Cross-sectional | The Kids' Inpatient Database | 28377 | 28377 (100) | 0 (0) | 14485 (51.1) | Yes | Yes | Parental income quartiles | Children and adults | Surgery |
| Meregaglia 2015 | Italy  2005 / 2008 / 2011 | Retrospective analysis of the national administrative database for IBD hospital admissions throughout Italy. | The Italian National Hospital Discharge Database | 109657 | 52166 (47.6) | 57491 (52.4) | 57708 (52.6) | Yes | No |  | Children and adults | Surgery |
| Peyrin-Biroulet 2012 | USA  Through March 2009 | Population-based inception cohort study | Medical records linkage system | 310 | 310 (100) | 0 (0) | 156 (50.3) | Yes | No |  | Children and adults | Surgery |
| Rinawi 2016 | Israel  1981 - 2013 | A retrospective analysis of medical charts | Medical charts and a national electronic database | 482 | 482 (100) | 0 (0) | 280 (58.1) | Yes | No |  | Only children (<18) | Surgery |
| Rinawi 2017 | Israel  1981- 2013 | Retrospective cohort, tertiary referral center | Medical charts and a national electronic database | 188 | 0 (0) | 188 (100) | 103 (54.8) | Yes | No |  | Only children (<18) | Surgery |
| Sato 2015 | Japan  1985 - 2010 | A single-center, retrospective, cohort study | A database of all CD patients treated at the Department of Gastroenterology, Fukuoka University Chikushi Hospital | 520 | 520 (100) | 0 (0) | 367 (70.6) | Yes | No |  | Children and adults | Surgery |
| Sceats 2019 | USA  2008 - 2014 | A retrospective cohort analysis | The 2007-2015 Truven MarketScan database | 38851 | 0 (0) | 38851 (100) | 18,648 (48) | Yes | No |  | Children and adults | Surgery |
| Solberg 2015 | Norway  1990 - 1994 | Prospective cohort, prediction model | Local hospital database | 519  (only 464 included in multi-variate analysis) | 0 (0) | 519 (100) | 267 (51.5) | Yes | No |  | Children and adults | Surgery |
| Stamatiou 2022 | UK  2000 - 2020 | Observational cohort study | Local electronic and paper healthcare records system | 1620 | 701 (43.3) | 919 (56.7) | 817 (50.4) | Yes | No |  | Adults | Surgery |
| Stokes 2018 | USA  2003, 2006, 2009, 2012 | Retrospective cohort | The Kid's Inpatient Database (KID), a nationally representative database | 20710 | 20710 (100) | 0 (0) | 10541 (50.9) | Yes | No |  | Children and adults | Surgery |
| Sun 2019 | China  Jan/2013 - Dec/2018 | Retrospective, observational, cohort study | Local hospital database | 246 | 246 (100) | 0 (0) | 149 (60.6) | Yes | No |  | Children and adults | Surgery |
| Tanaka 2021 | Japan  Aug/2008 - Mar/2020 | Retrospective cohort, outpatient clinic | Local hospital data | 95 | 0 (0) | 95 (100) | 61 (64.2) | Yes | No |  | Children and adults | Surgery |
| Targownik 2012 | Canada  1995 - Mar/2008 | Retrospective cohort | The University of Manitoba Inflammatory Bowel Disease Epidemiology Database, a population-based data-set | 3752 | 0 (0) | 3752 (100) | 1796 (47.9) | Yes | No |  | Adults | Surgery |
| Wan 2023 | China  Mar/2019 – Jan/2023 | Cross-sectional | The Chinese database for IBD (CHASE-IBD) | 8305 | 5077 (61.8) | 3140 (38.2) | 5245 (63.2) | Yes | No |  | Adults | Surgery |
| Wang 2022 | China  2016 – Nov/2021 | Retrospective cohort | National database | 1042 | 791 (75.9) | 251 (24.0) | 460 (44.1) | Yes | No |  | Adults | Surgery |
| Winder 2019 | Israel  2006 - 2014 | Retrospective cohort, single tertiary center | Medical records | 64 | 64 (100) | 0 (0) | 38 (59.4) | Yes | No |  | Adults | Surgery |
| Zhao 2019 | Denmark  Jan 2003 - Dec 2011 | Prospective inception cohort study | Danish National Patient Registry, Danish National Prescription Registry, Statistics Denmark | 213 | 213 (100) | 0 (0) | 99 (46.5) | Yes | No |  | Children and adults | Surgery |
| Timmer 2017 | Germany  2011/2012 and 2013 | Retrospective survey | Survey data; the German language pediatric IBD registry; epidemiological pediatric IBD registry of Saxony; patient lists of individual physicians and the national IBD patient organization. | 1280 | 804 (62.8) | 382 (29.8 / the remaining 94 (7.3%) were "Not specified") | 705 (55.1) | Yes | Yes | The highest education level, current occupation, and household income of the parents, divided into score quintiles (highest, middle categories, lowest) | Only children (<18); Children and adults | Surgery; Biologics |
| Lie 2017 | Netherlands  Mar/2006 - Feb/2011 | Prospective clinical cohort, but data on relevant outcomes for this review was cross-sectional, single tertiary center | Local data from the University Medical Center of Rotterdam | 188 | 188 (100) | 0 (0) | 81 (43.1) | Yes | No |  | Adults | Surgery ; Corticosteroids |
| Targownik 2014 | Canada  1987 - Mar/2010 | Retrospective cohort | Regional health insurance registry | 5300 | 2518 (47.5) | 2782 (52.5) | 2412 (45.5) | Yes | No |  | Children and adults | Surgery ; Corticosteroids |
| Dotson 2015 | USA  Apr/2004 - Jun/2012 | Cross-sectional | National administrative database | 5782 | 5782 (100) | 0 (0) | 2891 (50) | Yes | No |  | Only children (<18) | Surgery ; Corticosteroids ; Biologics |
| Herzog 2014 | Switzerland  2008 - Sep/2012 | Retrospective cohort | National database | 196 | 105 (53.6) | 91 (46.4) | 107 (54.6) | Yes | No |  | Only children (<18) | Surgery ; Corticosteroids ; Biologics |
| Liu 2022 | China  Jan/200 – Dec/2020 | Retrospective cohort | Electronic database | 611 | 611 (100) |  | 420 (69) | Yes | No |  | Adults | Surgery ; Corticosteroids ; Biologics |
| Gajendran 2016 | USA  2009 - 2011 | Cross-sectional study | Nationwide database | 113,993 | 78,16 (68.6) | 35,833 (31.4) | (45.7) | Yes | Yes | Low income (not further specified) | Adults | Surgery ; Hospitalization/inpatient visit |
| Gracie 2018 | UK  Sept/2014 - Jun/2017 | Longitudinal follow-up study | The original cross-sectional survey | 360 | 200 (55.6) | 160 (44.4) | - | Yes | No |  | Children and adults | Surgery ; Hospitalization/inpatient visit |
| Nguyen 2023 | USA  2010 - 2017 | Retrospective cohort | National health database | 1200 | 588 (49) | 612 (51) | 613 (51.1) | Yea | No |  | Adults | Surgery ; Hospitalization/inpatient visit |
| Osamura 2018 | Japan  Patients treated before Oct/2014 | Retrospective cohort | inclusion at Toho University, Sakura Medical Center | 219 | 219 (100) | 0 (0) | 165 (75) | Yes | No |  | Adults | Surgery ; Hospitalization/inpatient visit |
| Samuel 2013 | USA  1970 - 2006 | Retrospective cohort | Medical records | 369 | 0 (0) | 369 (100) | 216 (58) | Yes | No |  | Adults | Surgery ; Hospitalization/inpatient visit |
| Wong 2019 | USA  2007 - 2015 | Retrospective cohort | Truven Marketscan Commercial Claims and Encounters database | 331772 | 145610 (43.8) | 161336 (48.6) | 152283 (45.9) | Yes | No |  | Children and adults | Surgery ; Hospitalization/inpatient visit |
| Severs 2018 | The Netherlands  2010 - ? | Prospective cohorts, multicenter | Electronic database (surgery) and self-reported questionnaire data (medical treatment) | 3387 | 2118 (62.5) | 1269 (37.5) | male:female ratio: 1:1.7 (CD) / 1:1.1 (UC) | Yes | No |  | Adults | Surgery ; Hospitalization/inpatient visit ; Biologics |
| Bernstein 2020 | Canada  Apr/1995 - Mar/2018 | Population-based cohort study, province of Manitoba | Administrative (health) databases | 9298 | (47.3) | 0 (0) | 4240 (45.6) | No | Yes | Lower SES defined as (i) being registered for employment and income assistance, (ii) being registered with a service from Child and Family Services; or (iii) being in the highest Socioeconomic Factor Index quintile. | Adults | Surgery ; Hospitalization/inpatient visit ; Corticosteroids ; Biologics |
| McKenna 2018 | USA  Jan/2002 - Aug/2013 | Retrospective review | Prospectively maintained pouch database | 911 | 911 (100) | 0 (0) | 542 (59.5) | Yes | No |  | Adults | Surgery ; Hospitalization/inpatient visit ; Corticosteroids ; Biologics |
| Heath 2021 | Canada  Mar/2012 - Sept/2019 | Retrospective cohort study, University Personalized Medicine Clinic | Hospital records | 1015 | 656 (64.6) | 359 (35.4) | 456 (45) | Yes | No |  | Adults | Surgery ; Hospitalization/inpatient visit ; Corticosteroids ; Biologics |
