# Supplementary material for: Impact of sex and socioeconomic status on the likelihood of surgery, hospitalization, and use of medications in inflammatory bowel disease: a systematic review and meta-analysis
Source: Syst Rev. 2024 Jun 24;13:164. doi: 10.1186/s13643-024-02584-3 (PMC11194997; doi:10.1186/s13643-024-02584-3)
Supplement: Supplementary file 3 — Additional file 3: Table A3. Newcastle-Ottawa quality assessment. [file 13643_2024_2584_MOESM3_ESM.docx]

**Table A3. Newcastle-Ottawa quality assessment**

|  | **SELECTION** | | | | **COMPARABILITY** | | **OUTCOME** | | |  |  |
| --- | --- | --- | --- | --- | --- | --- | --- | --- | --- | --- | --- |
| Author, Year | Representativeness of the exposed cohort | Selection of the non-exposed cohort | Ascertainment of exposure | Demonstration that outcome of interest was not present at start of study | Study controls for the most important factors | Study controls for other factors | Assessment of outcome | Was follow-up long enough for outcomes to occur | Adequacy of follow up | **Total** | **Quality level*** |
| Chudy-Onwugaje 2021 | * | * | * | * | - | - | * | * | * | 7 | Fair |
| Gunnells 2015 | * | * | * | * | - | - | * | * | * | 7 | Fair |
| Heath 2021 | * | * | * | * | * | - | - | * | - | 6 | Fair |
| Li 2015 | * | * | * | * | * | * | * | - | - | 7 | Fair |
| Osamura 2018 | * | * | - | - | * | - | - | * | * | 5 | Fair |
| Solberg 2015 | * | * | * | * | - | - | * | * | * | 7 | Fair |
| Tanaka 2021 | * | * | * | * | - | - | * | * | * | 7 | Fair |
| AbouKhalil 2018 | * | * | * | * | * | - | * | * | * | 8 | Good |
| Axelrad 2019 | * | * | * | * | * | - | * | * | - | 7 | Good |
| Barnes 2017 | * | * | * | * | * | * | * | * | * | 9 | Good |
| Bernstein 2020 | * | * | * | * | * | * | * | * | * | 9 | Good |
| Chhay 2015 | * | * | * | * | * | * | * | * | * | 9 | Good |
| DeCristofaro 2022 | * | * | * | * | * | - | * | * | * | 8 | Good |
| Gao 2012 | * | * | * | - | * | - | * | * | * | 7 | Good |
| Goel 2013 | * | * | * | * | * | - | * | * | - | 7 | Good |
| Khalili 2020 | * | - | * | * | * | * | * | * | * | 8 | Good |
| Kim 2017 | * | * | * | - | * | * | * | * | * | 8 | Good |
| King 2020 | * | * | * | * | * | * | * | * | * | 9 | Good |
| Lagana 2019 | * | * | * | * | * | - | * | * | - | 7 | Good |
| Lee 2023 | * | * | * | * | * | - | * | * | * | 8 | Good |
| Lie 2017 | * | * | * | * | * | * | * | * | * | 9 | Good |
| Limsrivilai 2017 | * | * | * | * | * | * | * | * | * | 9 | Good |
| Lin 2013 | * | * | * | - | * | - | * | * | * | 7 | Good |
| Liu 2022 | * | * | * | * | * | - | * | * | * | 8 | Good |
| Mandel 2014 | * | * | * | * | * | - | * | * | - | 7 | Good |
| McKenna 2018 | * | * | * | * | * | * | * | * | * | 9 | Good |
| Meregaglia 2015 | * | * | * | - | * | * | * | * | - | 7 | Good |
| Micic 2017 | * | * | * | * | * | - | * | * | - | 7 | Good |
| Mudireddy 2017 | * | * | * | * | * | * | * | * | * | 9 | Good |
| Nguyen 2023 | * | * | * | * | * | * | * | * | * | 9 | Good |
| Peyrin-Biroulet 2012 | * | * | * | * | * | - | * | * | * | 8 | Good |
| Poojary 2017 | * | * | * | * | * | * | * | * | - | 8 | Good |
| Reja 2020 | - | * | * | * | * | * | * | * | - | 7 | Good |
| Rinawi 2016 | * | * | * | * | * | * | * | * | - | 8 | Good |
| Rinawi 2017 | * | * | * | * | * | * | * | * | * | 9 | Good |
| Rundquist 2018 | * | * | * | * | * | * | * | * | * | 9 | Good |
| Samuel 2013 | * | * | * | * | * | - | * | - | * | 7 | Good |
| Sato 2015 | * | * | * | * | * | - | * | * | - | 7 | Good |
| Sceats 2019 | * | * | * | * | * | * | * | * | * | 9 | Good |
| Schultheiss 2019 | * | * | * | * | * | * | * | * | * | 9 | Good |
| Stamatiou 2022 | * | * | * | * | * | - | * | * | * | 8 | Good |
| Stokes 2018 | * | * | * | - | * | * | * | * | * | 8 | Good |
| Sun 2019 | * | * | * | * | * | * | * | * | * | 9 | Good |
| Tanaka 2018 | * | * | * | * | * | * | * | * | - | 8 | Good |
| Targownik 2012 | * | * | * | * | * | - | * | * | * | 8 | Good |
| Targownik 2014 | * | * | * | - | * | - | * | * | * | 7 | Good |
| Wang 2022 | * | * | * | * | * | - | * | * | * | 8 | Good |
| Wong 2019 | * | * | * | * | * | * | * | * | - | 8 | Good |
| Zhao 2019 | * | * | * | * | * | * | * | * | * | 9 | Good |
| Akintimehin 2018 | * | * | * | - | - | - | * | * | * | 6 | Poor |
| Gracie 2018 | * | * | * | - | - | - | * | * | - | 5 | Poor |
| Severs 2018 | * | * | * | * | - | - | * | - | * | 6 | Poor |
| Winder 2019 | * | * | * | * | - | - | * | * | - | 6 | Poor |
| Barkan 2024 | - | - | - | - | - | - | - | - | - | -^a^ |  |
| Calvo-Arbeloa 2020 | - | - | - | - | - | - | - | - | - | -^a^ |  |
| daSilva 2015 | - | - | - | - | - | - | - | - | - | -^a^ |  |
| Dotson 2015 | - | - | - | - | - | - | - | - | - | -^a^ |  |
| Eder 2017 | - | - | - | - | - | - | - | - | - | -^a^ |  |
| Gajendran 2016 | - | - | - | - | - | - | - | - | - | -^a^ |  |
| Herzog 2014 | - | - | - | - | - | - | - | - | - | -^a^ |  |
| Lee 2012 | - | - | - | - | - | - | - | - | - | -^a^ |  |
| Magro 2019 | - | - | - | - | - | - | - | - | - | -^b^ |  |
| Mahlich 2018 | - | - | - | - | - | - | - | - | - | -^a^ |  |
| McLoughlin 2020 | - | - | - | - | - | - | - | - | - | -^a^ |  |
| Sundel 2022 | - | - | - | - | - | - | - | - | - | -^a^ |  |
| Timmer 2017 | - | - | - | - | - | - | - | - | - | -^a^ |  |
| Wan 2023 | - | - | - | - | - | - | - | - | - | -^a^ |  |

^*^ ^Good quality: 3 or 4 stars in selection domain AND 1 or 2 stars in comparability domain AND 2 or 3 stars in outcome/exposure domain; Fair quality: 2 stars in selection domain AND 1 or 2 stars in comparability domain AND 2 or 3 stars in outcome/exposure domain; Poor quality: 0 or 1 star in selection domain OR 0 stars in comparability domain OR 0 or 1 stars in outcome/exposure domain^

^a^ Cross sectional study or cross sectional data collection for relevant outcome

^b^ Prediction model
