# Supplementary material for: Impact of sex and socioeconomic status on the likelihood of surgery, hospitalization, and use of medications in inflammatory bowel disease: a systematic review and meta-analysis
Source: Syst Rev. 2024 Jun 24;13:164. doi: 10.1186/s13643-024-02584-3 (PMC11194997; doi:10.1186/s13643-024-02584-3)
Supplement: Supplementary file 4 — Additional file 4: Table A4. GRADE evidence profile of risk of surgery and hospitalization. [file 13643_2024_2584_MOESM4_ESM.docx]

**Table A4. GRADE evidence profile of risk of surgery and hospitalization**

| **Outcome** | **Study design** | **Risk of bias** | **Inconsistency** | **Indirectness** | **Imprecision** | **Publication bias** | **Other considerations** | **Quality** |
| --- | --- | --- | --- | --- | --- | --- | --- | --- |
| Surgery | Observational | Low | High heterogeneity | Indirectness as most studies did not examine sex or SES differences as the primary aim | Risk of imprecision as the pooled estimates had large standard errors/broad confidence intervals | No serious publication bias | None | Very low |
| Hospitalization | Observational | Low | High heterogeneity | Indirectness as most studies did not examine sex or SES differences as the primary aim | Risk of imprecision as the pooled estimates had large standard errors/broad confidence intervals | No serious publication bias | None | Very low |

The quality of evidence of the studies for both surgery and hospitalization was graded low due to the observational study designs, and further downgraded to very low due to a high inconsistency and indirectness.
