# Supplementary material for: Impact of sex and socioeconomic status on the likelihood of surgery, hospitalization, and use of medications in inflammatory bowel disease: a systematic review and meta-analysis
Source: Syst Rev. 2024 Jun 24;13:164. doi: 10.1186/s13643-024-02584-3 (PMC11194997; doi:10.1186/s13643-024-02584-3)
Supplement: Supplementary file 5 — Additional file 5: Figure A1. Meta-regression of log HRs for risk of surgery by study publication year. Figure A2. Meta-regression of log ORs for risk of hospitalization by study publication year. Figure A3. Forest plot of subgroup (univariate/multivariate) meta-analysis of HRs for risk of surgery. Figure A4. Forest plot of subgroup (univariate/multivariate) meta-analysis of ORs for risk of hospitalization. Figure A5. Funnel plot of the meta-analysis of published studies on surgery. Figure A6. Funnel plot of the meta-analysis of published studies on hospitalization. [file 13643_2024_2584_MOESM5_ESM.docx]

**Additional file A5 - figures**

**Meta-regression**


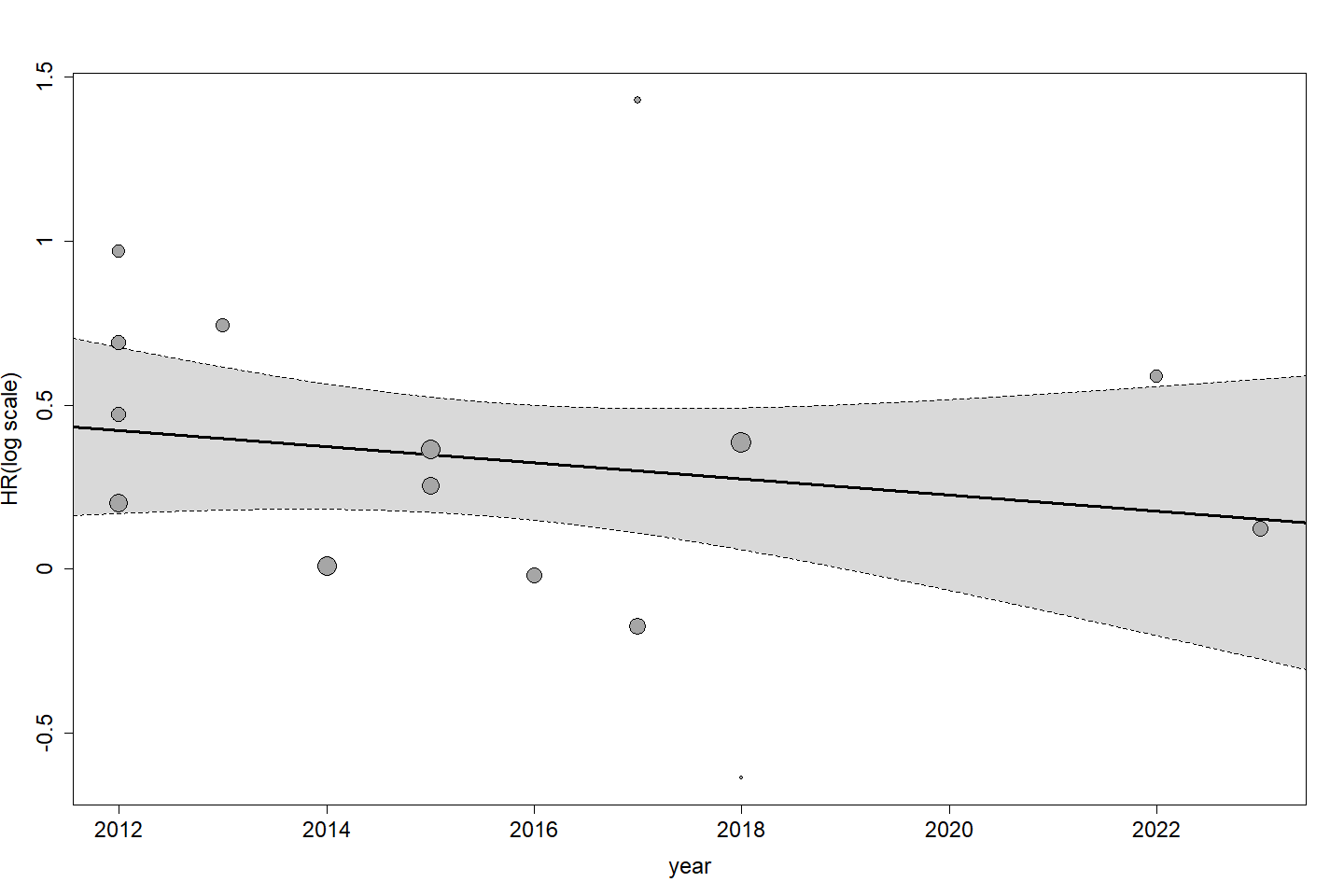


**Figure A1. Meta-regression of log HRs for risk of surgery by study publication year**

Meta-regression of log HRs for risk of surgery in male patients compared with female patients with IBD by study publication year. Reference: female patients. Abbreviations: HR, hazard ratio; IBD, inflammatory bowel disease


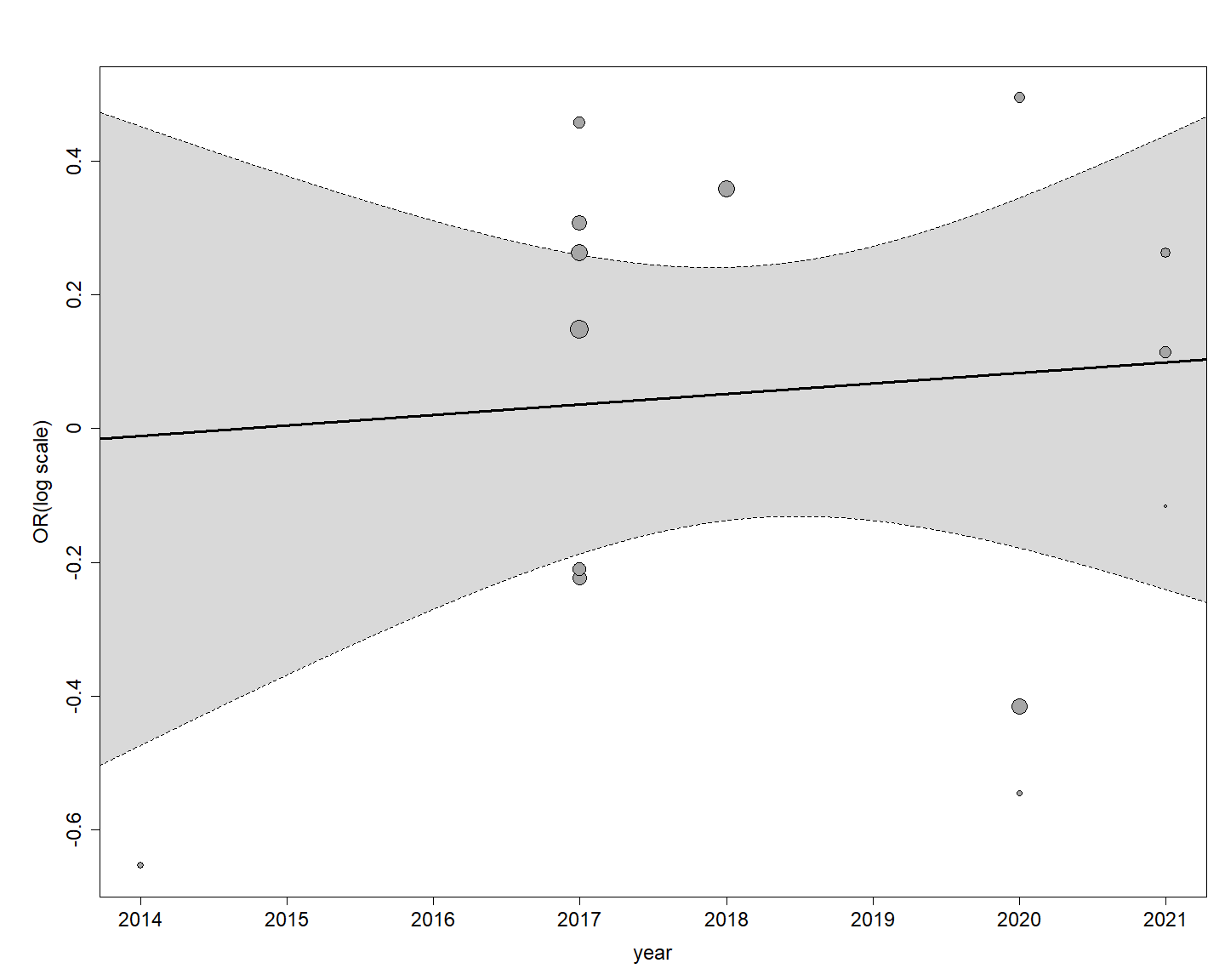


**Figure A2. Meta-regression of log ORs for risk of hospitalization by study publication year**

Meta-regression of log ORs for risk of hospitalization in male patients compared with female patients with IBD by study publication year. Reference: female patients. Abbreviations: OR, odds ratio; IBD, inflammatory bowel disease

**Sensitivity analyses and publication bias**


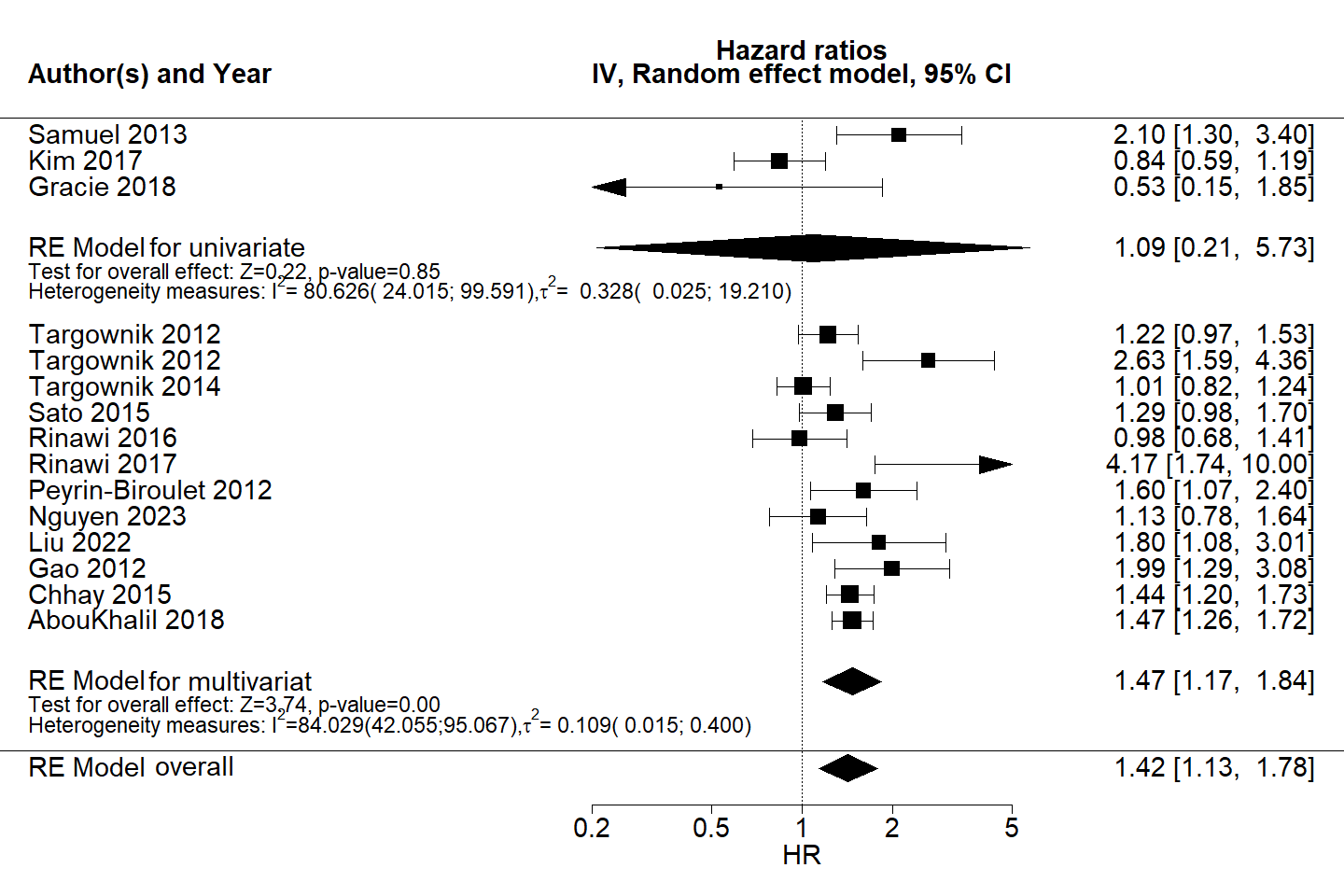


**Figure A3. Forest plot of subgroup (univariate/multivariate) meta-analysis of HRs for risk of surgery**

Forest plot of RE model subgroup meta-analysis of HRs for risk of surgery in male patients compared with female patients with IBD by univariate/multivariate subgroups. Reference: female patients. Abbreviations: HR, hazard ratio; IBD, inflammatory bowel disease; NA, not applicable; RE, random effects

**
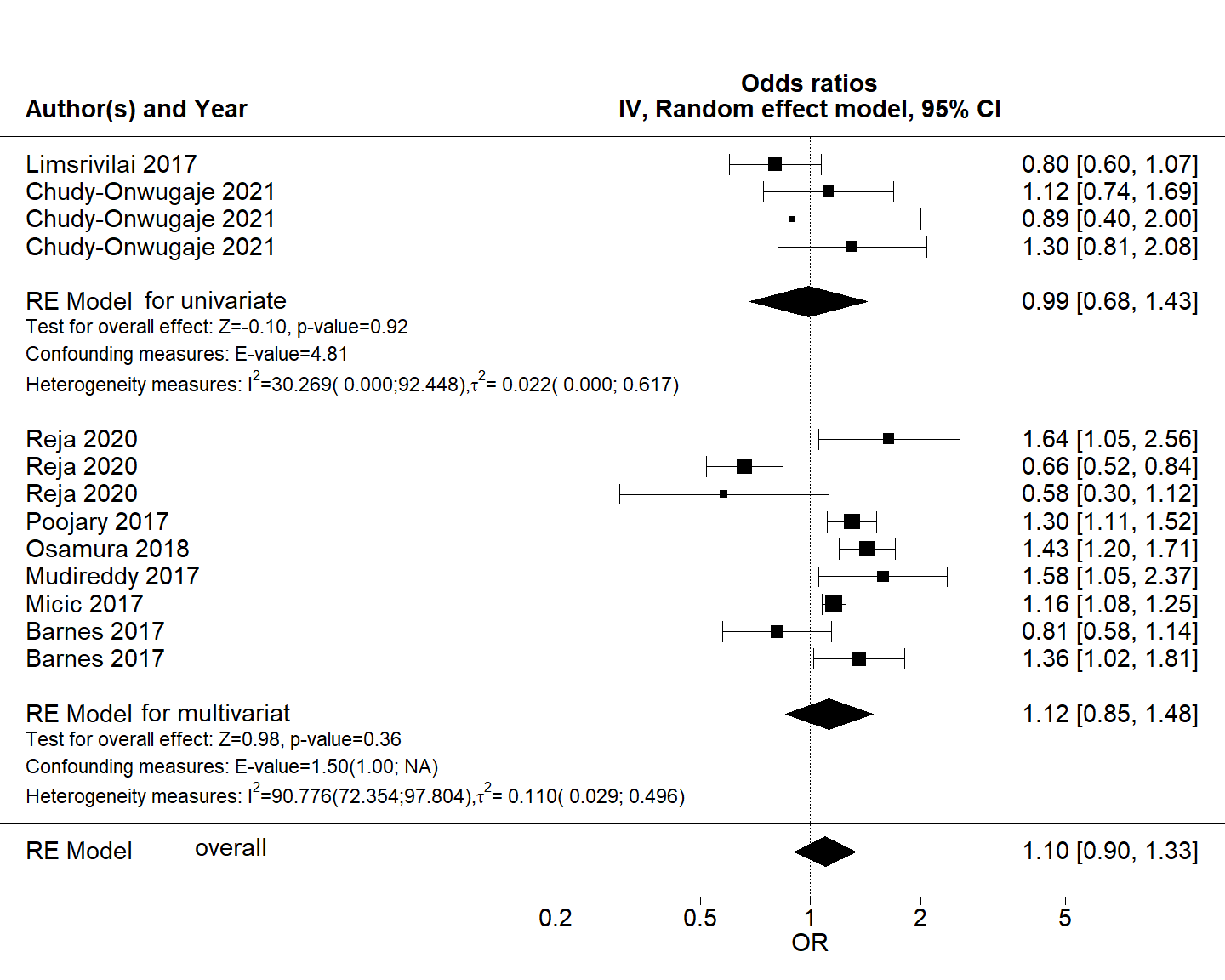
**

**Figure A4. Forest plot of subgroup (univariate/multivariate) meta-analysis of ORs for risk of hospitalization**

Forest plot of RE model subgroup meta-analysis of ORs for risk of hospitalization in male patients compared with female patients with IBD by univariate/multivariate subgroups. Reference: female patients. Abbreviations: OR, odds ratio; IBD, inflammatory bowel disease; NA, not applicable; RE, random effects

**
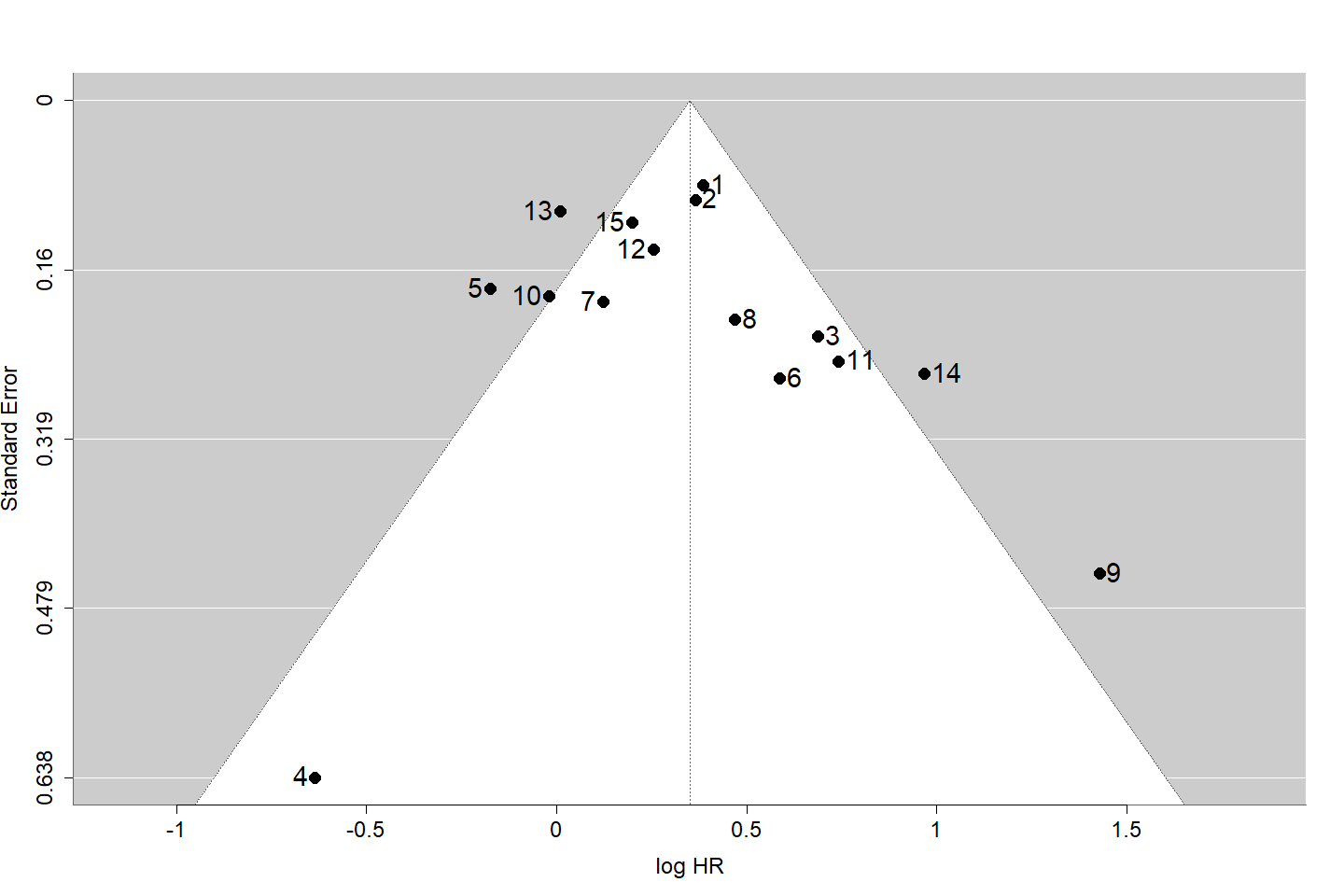
**

**Figure A5. Funnel plot of the meta-analysis of published studies on surgery**

Each plotted point represents the standard error and log(HR) for male patients compared to female patients for a single study. The white triangle represents the region where 95% of the data points would lie in the absence of publication bias. The vertical line represents the average log(HR) estimate found in the meta-analysis. Abbreviations: HR, hazard ratio.

1: AbouKhalil 2018; 2: Chhay 2015; 3: Gao 2012; 4: Gracie 2018; 5: Kim 2017; 6: Liu 2022; 7: Nguyen 2023; 8: Peyrin-Biroulet 2012; 9: Rinawi 2017; 10: Rinawi 2016; 11: Samuel 2013; 12: Sato 2015; 13: Targownik 2014; 14: Targownik 2012; 15: Targownik 2012


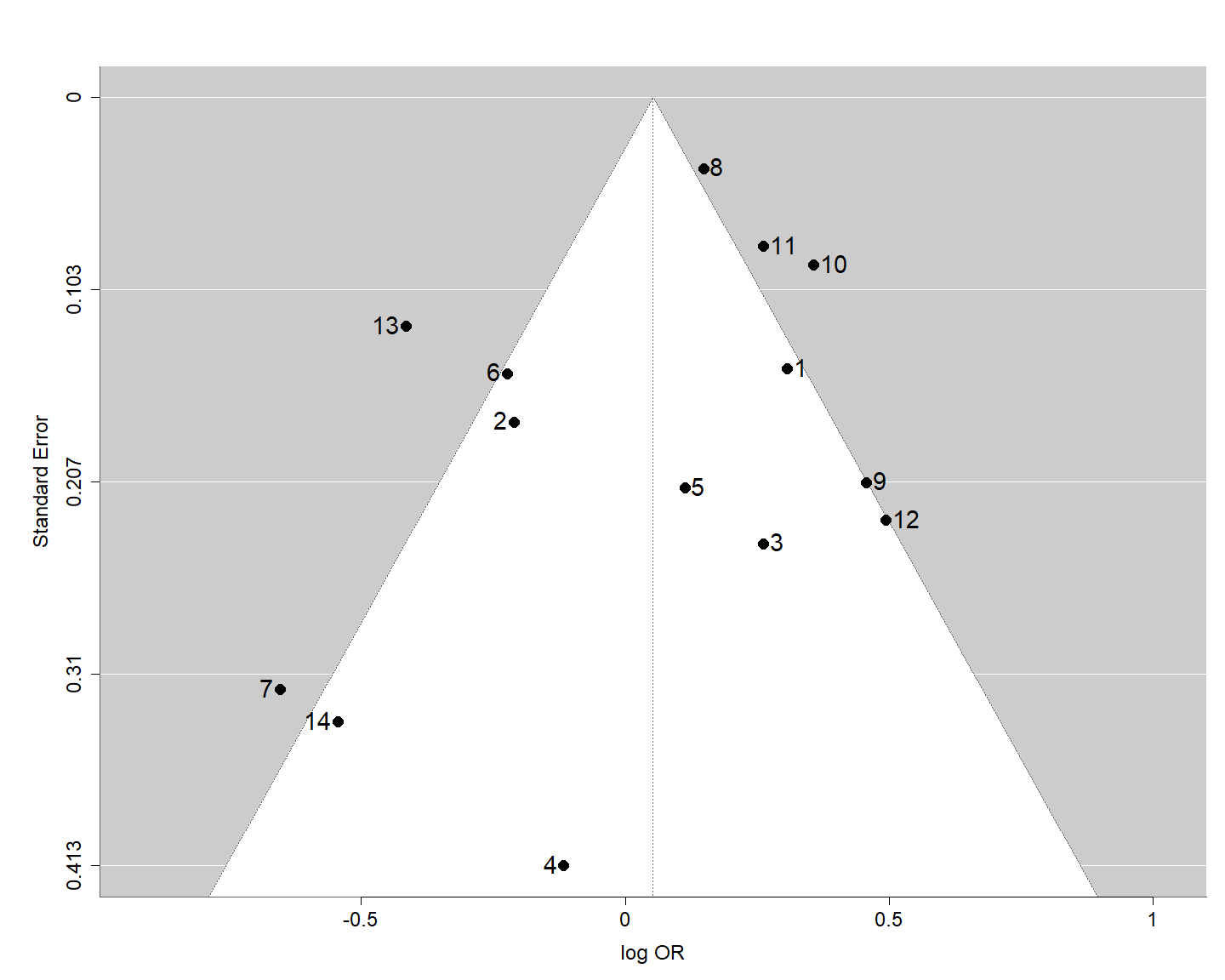


**Figure A6. Funnel plot of the meta-analysis of published studies on hospitalization**

Each plotted point represents the standard error and log(OR) for male patients compared to female patients for a single study. The white triangle represents the region where 95% of the data points would lie in the absence of publication bias. The vertical line represents the average log(OR) estimate found in the meta-analysis. Abbreviations: OR, odds ratio.

1: Barnes 2017; 2: Barnes 2017; 3: Chudy-Onwugaje 2021; 4: Chudy-Onwugaje 2021; 5: Chudy-Onwugaje 2021; 6: Limsrivilai 2017; 7: Mandel 2014; 8: Micic 2017; 9: Mudireddy 2017; 10: Osamura 2018; 11: Poojary 2017; 12: Reja 2020; 13: Reja 2020; 14: Reja 2020; 15: Reja 2020
